# Supplementary material for: Novel Arylsulfonylhydrazones as Breast Anticancer Agents Discovered by Quantitative Structure-Activity Relationships
Source: Molecules. 2023 Feb 22;28(5):2058. doi: 10.3390/molecules28052058 (PMC10004090; doi:10.3390/molecules28052058)
Supplement: Supplementary file 1 [file molecules-28-02058-s001.zip › molecules-2202805-supplementary.pdf]

Article

Novel Arylsulfonylhydrazones as Breast Anticancer Agents Discovered by Quantitative Structure-Activity Relationships

Violina T. Angelova [1,*], Teodora Tatarova [1], Rositsa Mihaylova [1], Nikolay Vassilev [2], Boris Petrov [1], Zvetanka Zhivkova [1] and Irini Doytchinova [1,*]

[1] Faculty of Pharmacy, Medical University of Sofia, 1000 Sofia, Bulgaria
[2] Laboratory "Nuclear Magnetic Resonance", Institute of Organic Chemistry with Centre of Phytochemistry, Bulgarian Academy of Sciences, 1113 Sofia, Bulgaria
* Correspondence: v.stoyanova@pharmfac.mu-sofia.bg (V.T.A.); idoytchinova@pharmfac.mu-sofia.bg (I.D.)

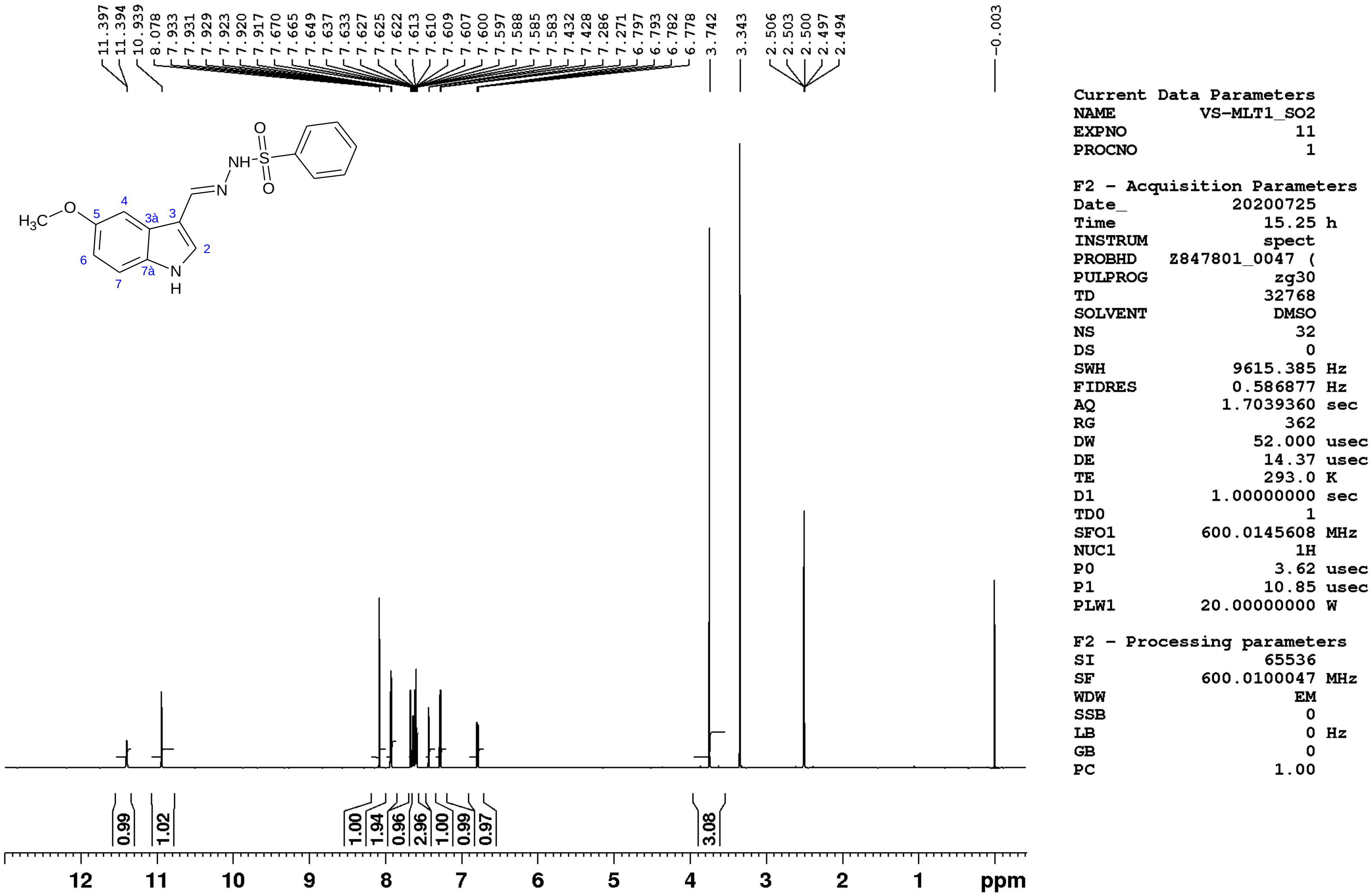

Figure S1. ^{1}H NMR spectrum of *N'*-[(*E*)-(5-methoxy-1*H*-indol-3-yl)methylidene]benzenesulfonohydrazide, **1a** in DMSO-d_6

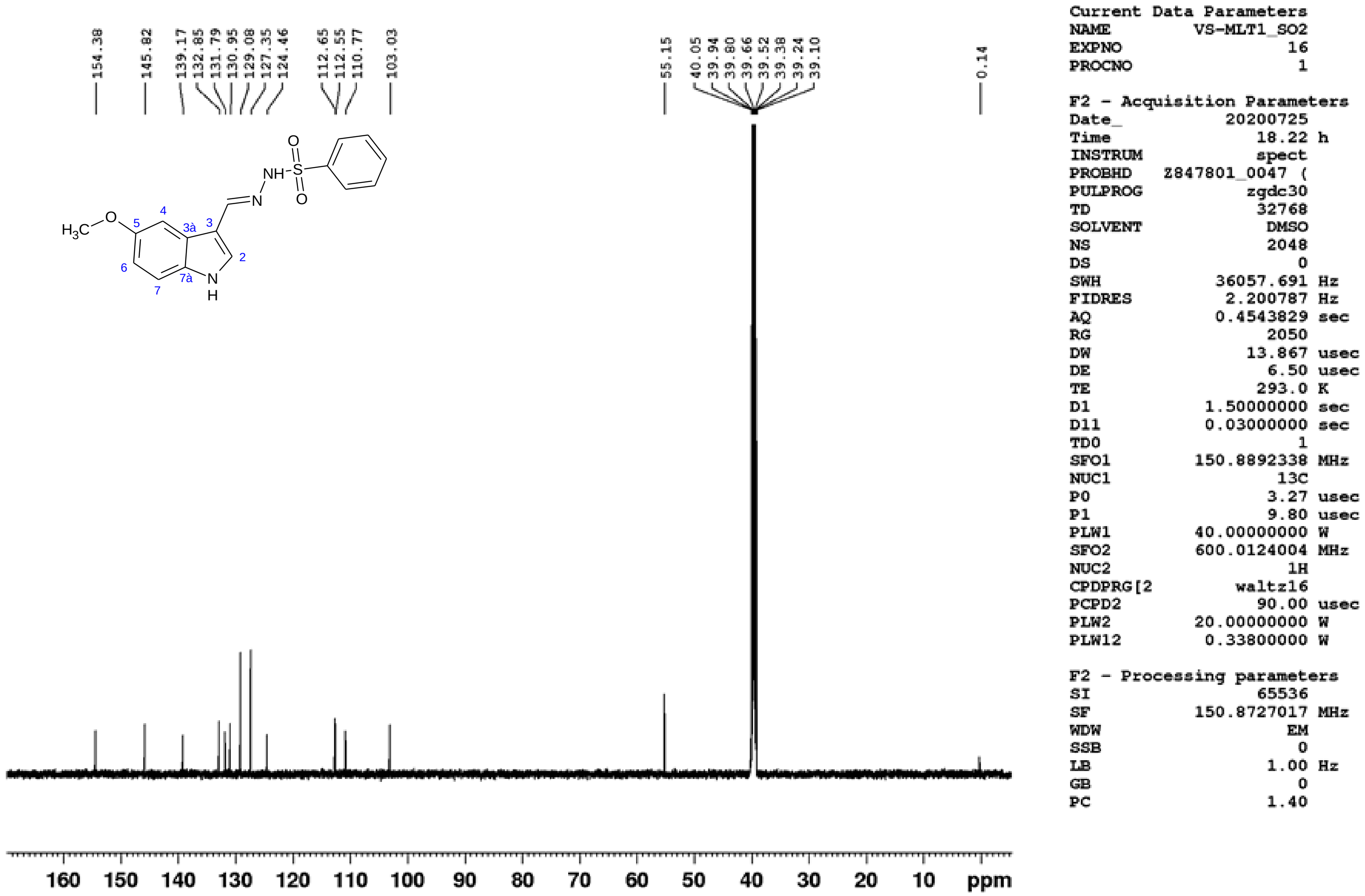

Figure S2. ^{13}C NMR spectrum of *N'*-[(*E*)-(5-methoxy-1*H*-indol-3-yl)methylidene]benzenesulfonohydrazide, **1a** in DMSO-d_6

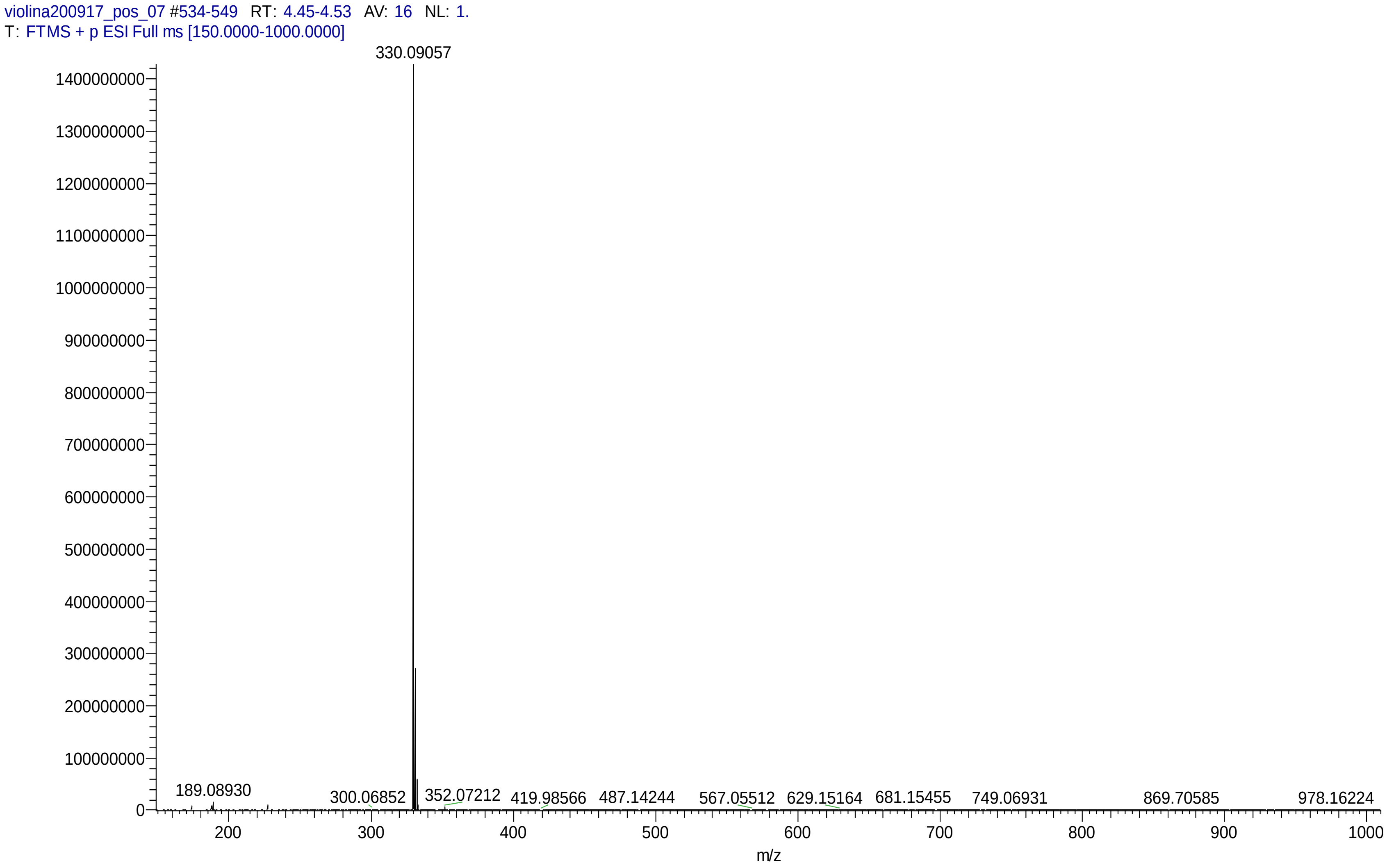

Figure S3. HRMS of *N*'-[(*E*)-(5-methoxy-1*H*-indol-3-yl)methylidene]benzenesulfonohydrazide, **1a**

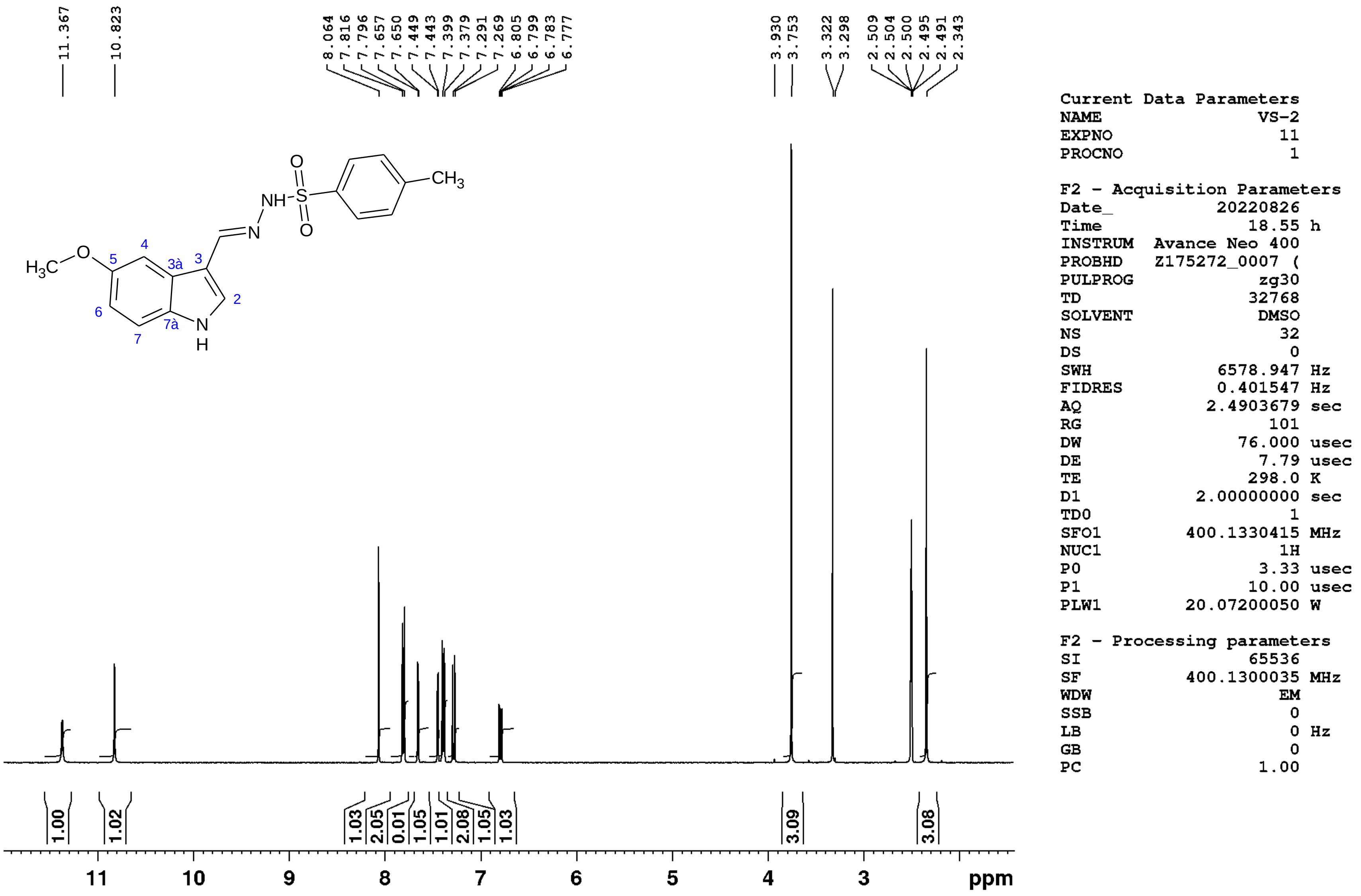

Figure S4. ^{1}H NMR spectrum of *N'*-[(*E*)-(5-methoxy-1*H*-indol-3-yl)methylidene]-4-methylbenzenesulfonohydrazide, **1b** in DMSO-d_6

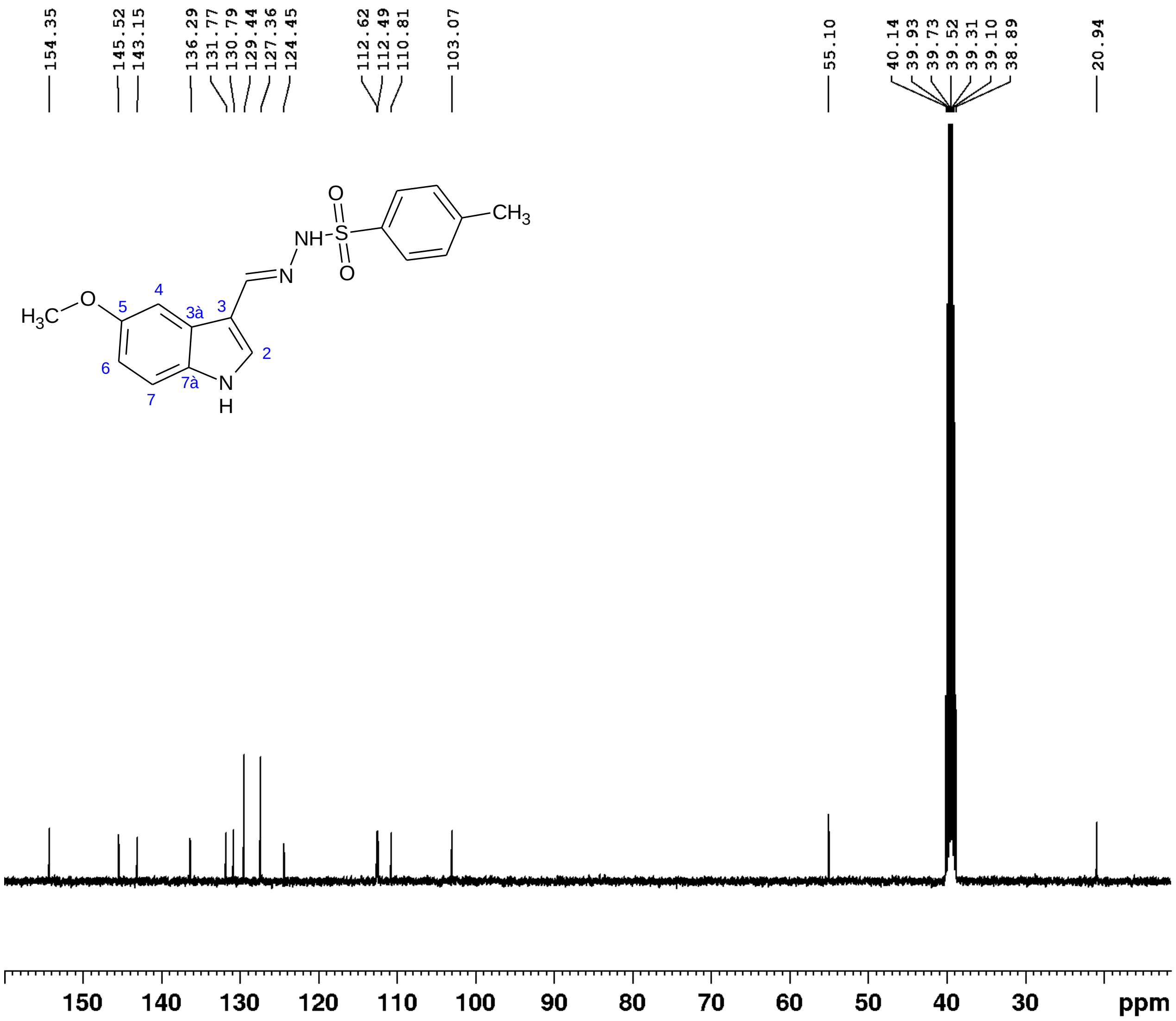

Current Data Parameters
NAME VS-2
EXPNO 17
PROCNO 1

F2 - Acquisition Parameters
Date_ 20220827
Time 4.07 h
INSTRUM Avance Neo 400
PROBHD Z175272_0007 (
PULPROG zgdc30
TD 32768
SOLVENT DMSO
NS 1024
DS 0
SWH 23809.523 Hz
FIDRES 1.453218 Hz
AQ 0.6881280 sec
RG 32
DW 21.000 usec
DE 6.50 usec
TE 298.0 K
D1 1.50000000 sec
D11 0.03000000 sec
TD0 1
SFO1 100.6238383 MHz
NUC1 13C
P0 3.33 usec
P1 10.00 usec
PLW1 58.46900177 W
SFO2 400.1316008 MHz
NUC2 1H
CPDPRG[2 waltz16
PCPD2 90.00 usec
PLW2 20.07200050 W
PLW12 0.24781001 W

F2 - Processing parameters
SI 65536
SF 100.6128172 MHz
WDW EM
SSB 0
LB 1.00 Hz
GB 0
PC 1.40

Figure S5. ^{13}C NMR spectrum of *N*'-[(*E*)-(5-methoxy-1*H*-indol-3-yl)methylidene]-4-methylbenzenesulfonohydrazide, **1b** in DMSO-d_6

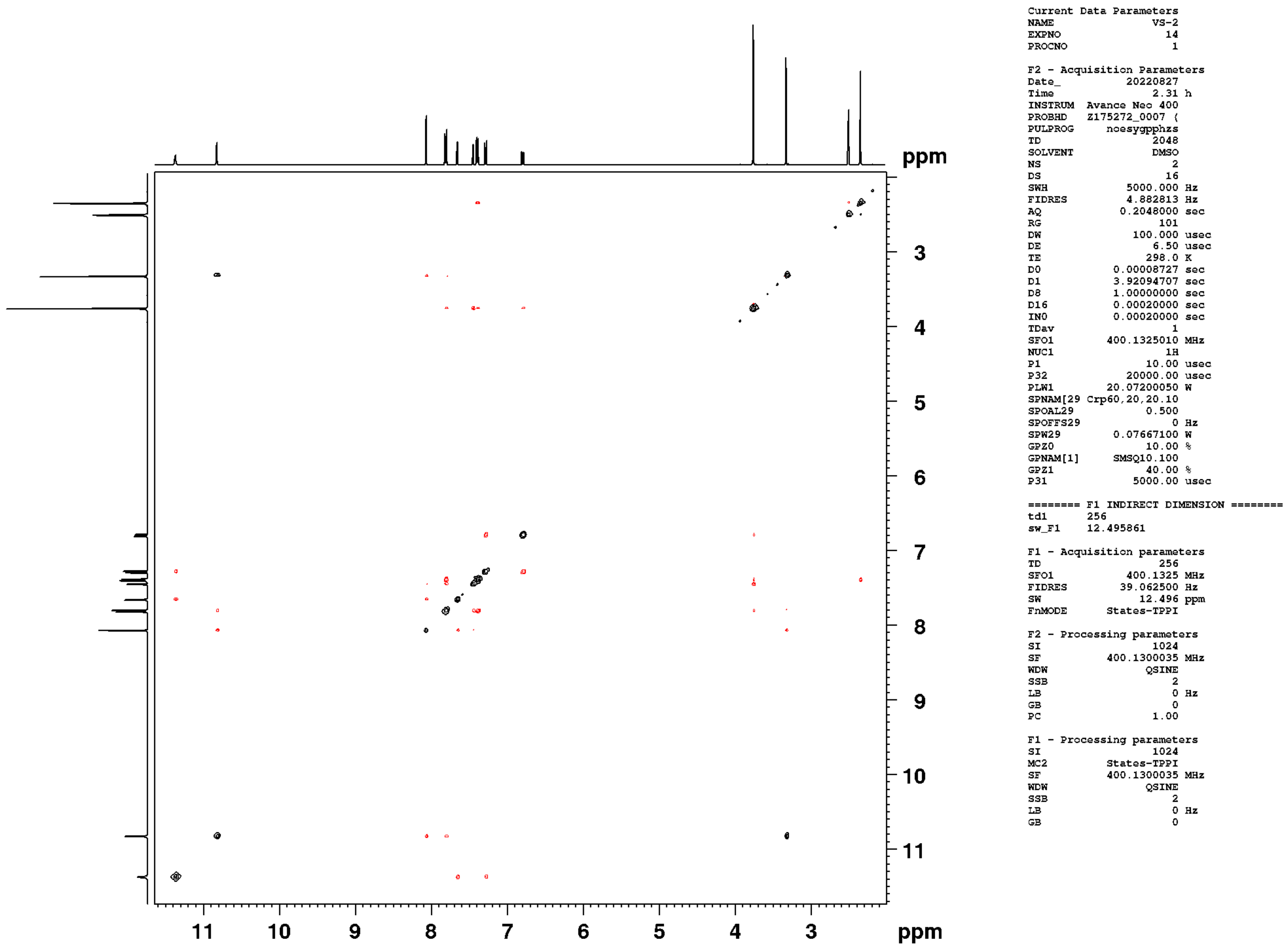

Figure S6. NOESY spectrum of *N'*-[(*E*)-(5-methoxy-1*H*-indol-3-yl)methylidene]-4-methylbenzenesulfonohydrazide, **1b** in DMSO-d_6

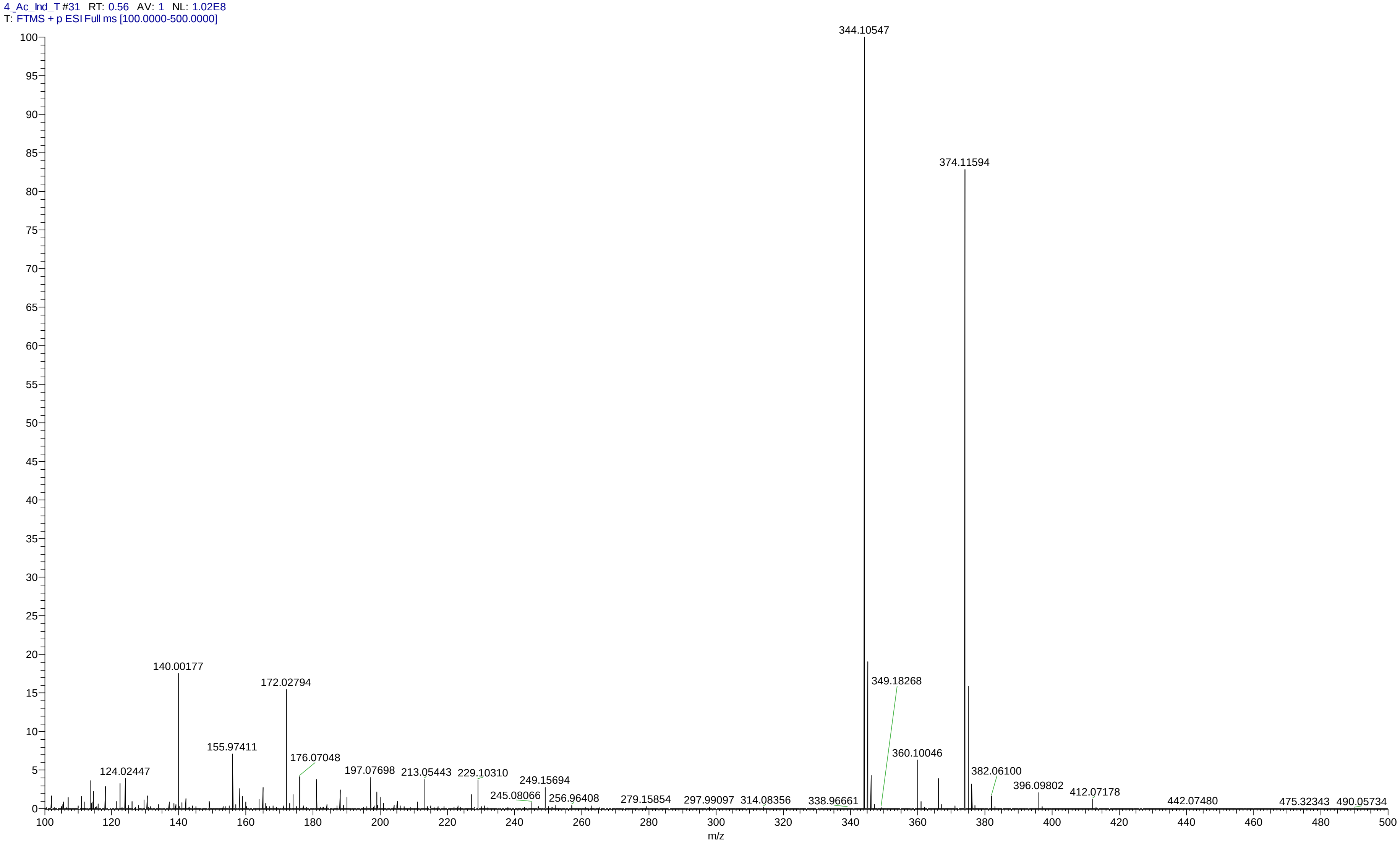

Figure S7. HRMS of *N'*-[(*E*)-(5-methoxy-1*H*-indol-3-yl)methylidene]-4-methylbenzenesulfonohydrazide, **1b**

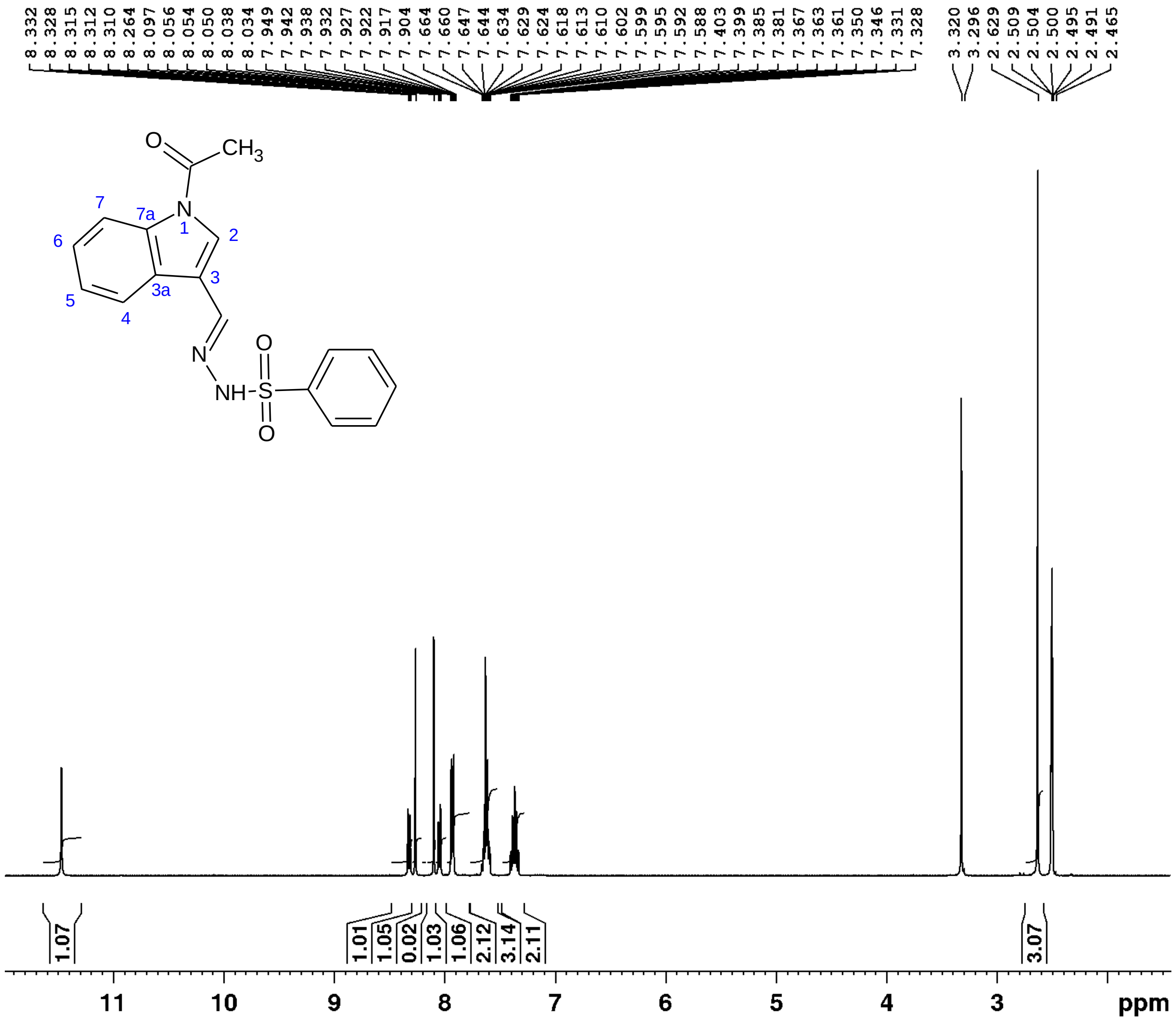

```
Current Data Parameters
NAME               VS-3
EXPNO                11
PROCNO                1

F2 - Acquisition Parameters
Date_          20220826
Time              19.05 h
INSTRUM   Avance Neo 400
PROBHD   Z175272_0007 (
PULPROG            zg30
TD                32768
SOLVENT            DMSO
NS                   32
DS                    0
SWH            6578.947 Hz
FIDRES         0.401547 Hz
AQ            2.4903679 sec
RG                  101
DW               76.000 usec
DE                 7.79 usec
TE                298.0 K
D1           2.00000000 sec
TD0                   1
SFO1        400.1330415 MHz
NUC1                 1H
P0                 3.33 usec
P1                10.00 usec
PLW1        20.07200050 W

F2 - Processing parameters
SI                65536
SF          400.1300035 MHz
WDW                  no
SSB                   0
LB                    0 Hz
GB                    0
PC                 1.00
```

Figure S8. ^{1}H NMR spectrum of *N*'-[(*E*)-(1-acetyl-1*H*-indol-3-yl)methylidene]benzenesulfonohydrazide, **1c** in DMSO-d_6

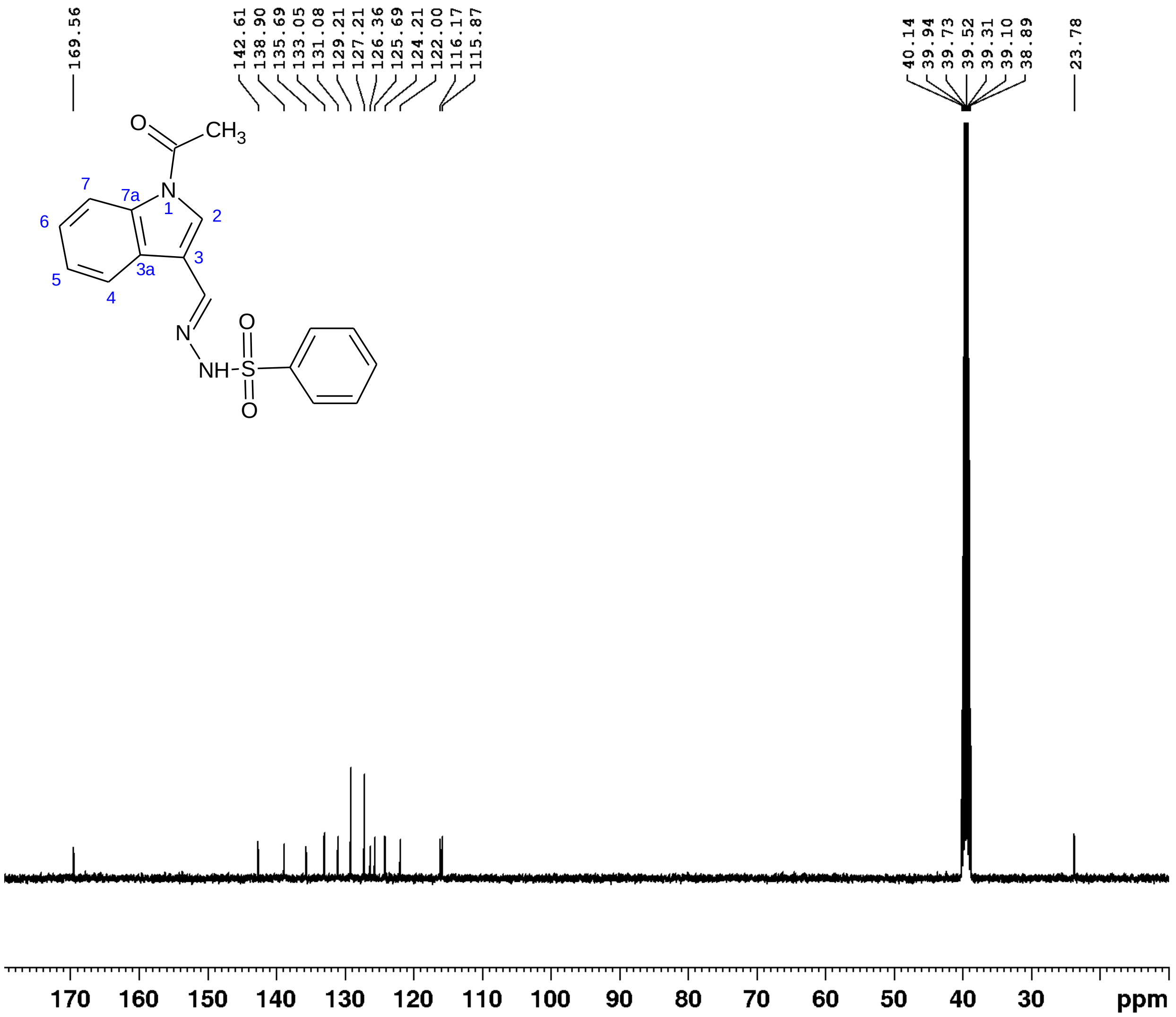


```
Current Data Parameters
NAME                VS-3
EXPNO                 17
PROCNO                 1

F2 - Acquisition Parameters
Date_           20220827
Time                6.53 h
INSTRUM   Avance Neo 400
PROBHD    Z175272_0007 (
PULPROG          zgdc30
TD                32768
SOLVENT             DMSO
NS                  1024
DS                     0
SWH            23809.523 Hz
FIDRES          1.453218 Hz
AQ             0.6881280 sec
RG                    32
DW                21.000 usec
DE                  6.50 usec
TE                 298.0 K
D1            1.50000000 sec
D11           0.03000000 sec
TD0                    1
SFO1         100.6238383 MHz
NUC1                 13C
P0                  3.33 usec
P1                 10.00 usec
PLW1         58.46900177 W
SFO2         400.1316008 MHz
NUC2                  1H
CPDPRG[2         waltz16
PCPD2              90.00 usec
PLW2         20.07200050 W
PLW12         0.24781001 W

F2 - Processing parameters
SI                 65536
SF           100.6128174 MHz
WDW                   EM
SSB                    0
LB                  1.00 Hz
GB                     0
PC                  1.40
```

Figure S9. ^{13}C NMR spectrum of *N*'-[(*E*)-(1-acetyl-1*H*-indol-3-yl)methylidene]benzenesulfonohydrazide, **1c** in DMSO-d_6

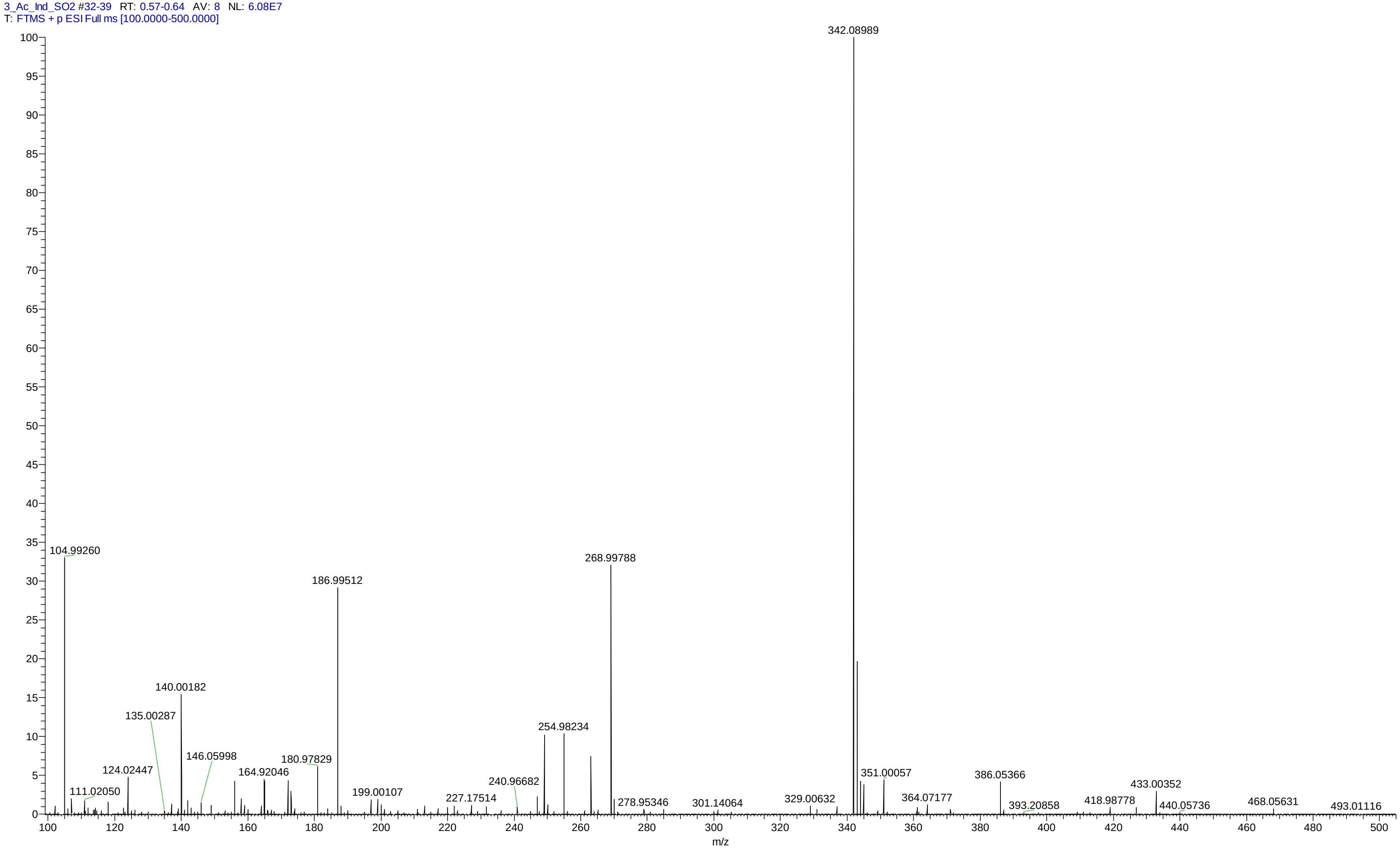

Figure S10. HRMS of *N*'-[(*E*)-(1-acetyl-1*H*-indol-3-yl)methylidene]benzenesulfonohydrazide, **1c**

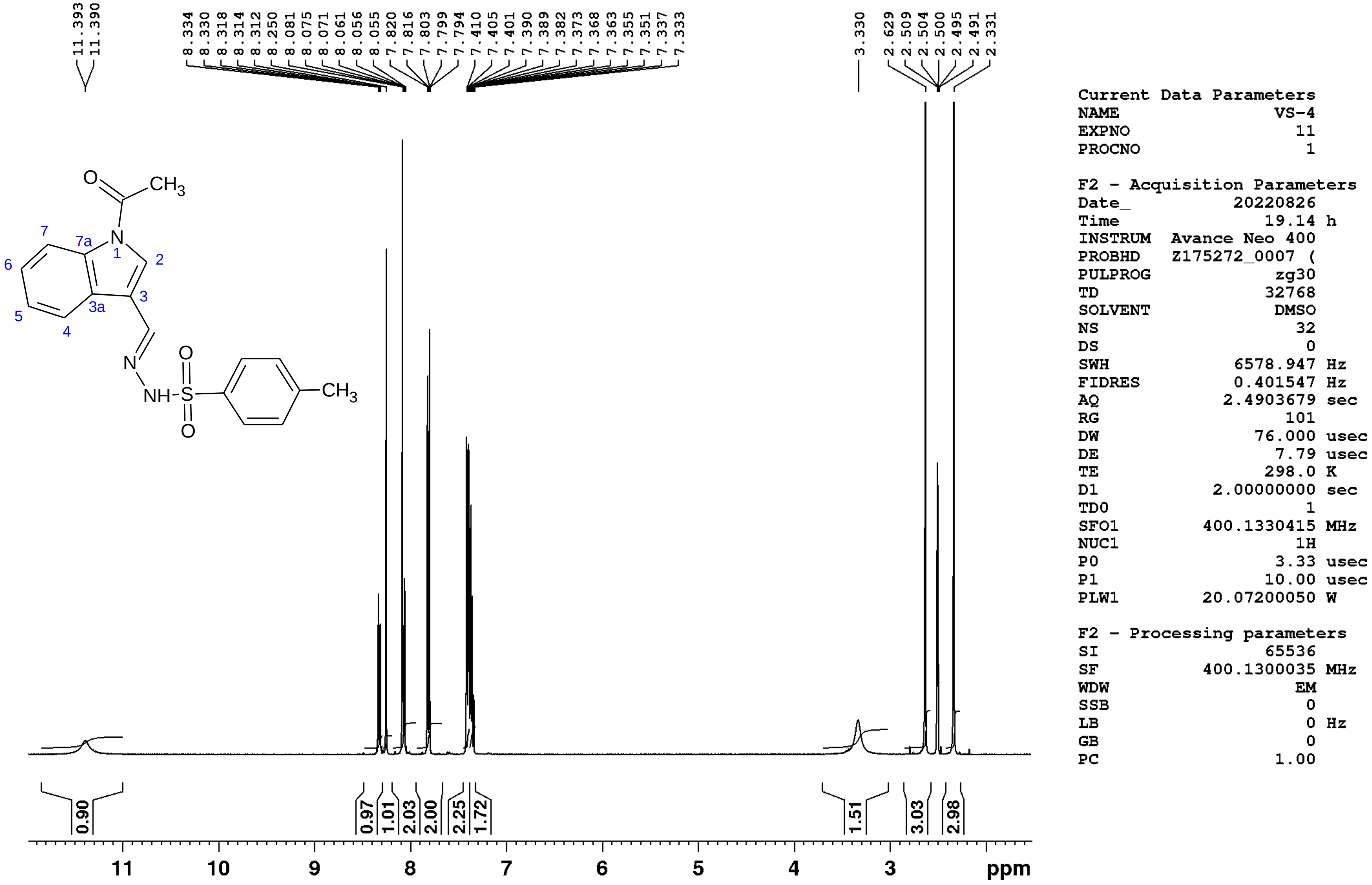

Figure S11. ^{1}H NMR spectrum of *N'*-[(*E*)-(1-acetyl-1*H*-indol-3-yl)methylidene]-4-methylbenzenesulfonohydrazide, **1d** in DMSO-d_6

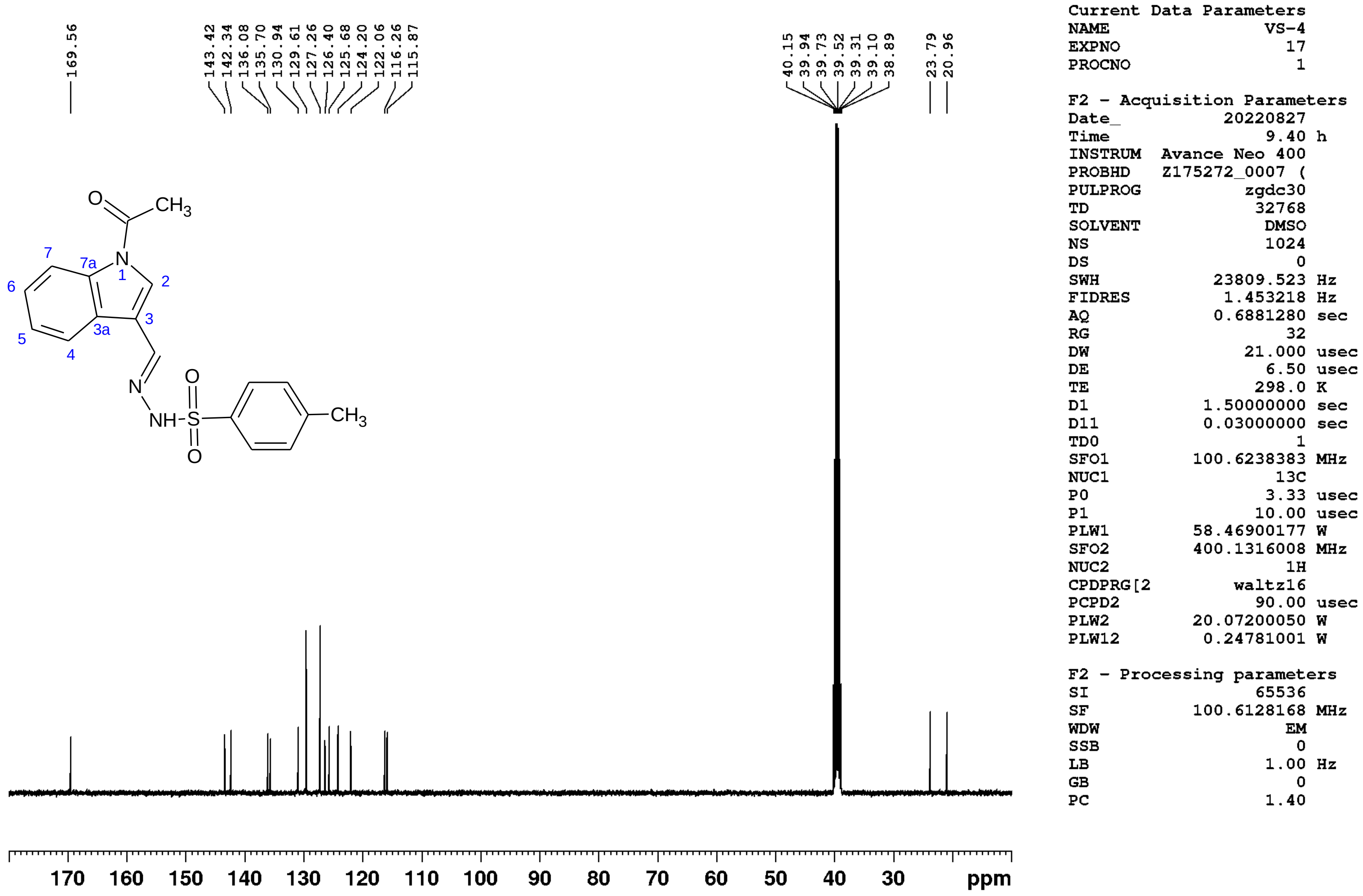

Figure S12. ^{13}C NMR spectrum of *N*'-[(*E*)-(1-acetyl-1*H*-indol-3-yl)methylidene]-4-methylbenzenesulfonohydrazide, **1d** in DMSO-d_6

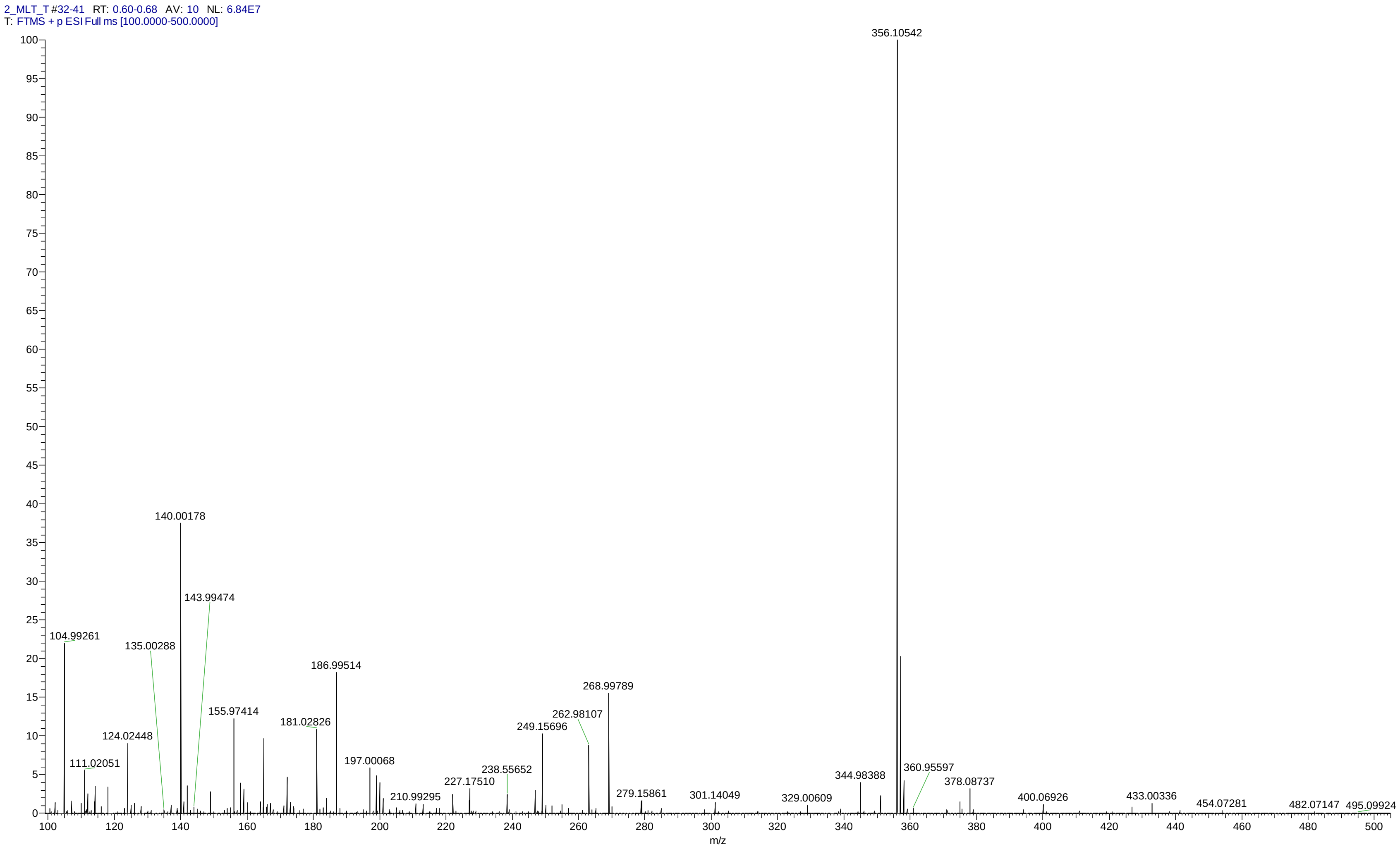

Figure S13. HRMS of *N*'-[(*E*)-(1-acetyl-1*H*-indol-3-yl)methylidene]-4-methylbenzenesulfonohydrazide, **1d**

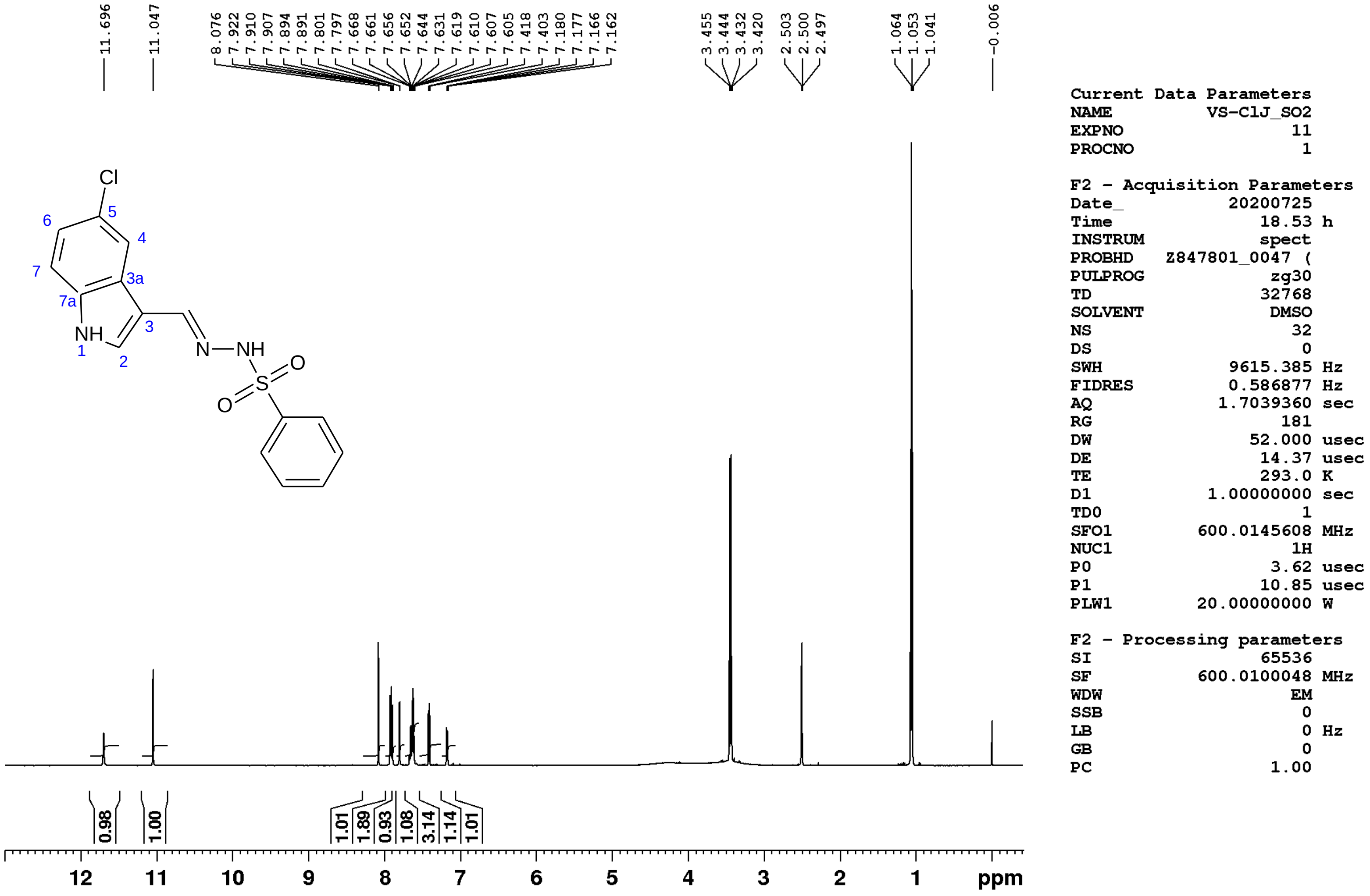

Figure S14. ^{1}H NMR spectrum of *N'*-[(*E*)-(5-chloro-1*H*-indol-3-yl)methylidene]benzenesulfonohydrazide, **1e** in DMSO-d_6

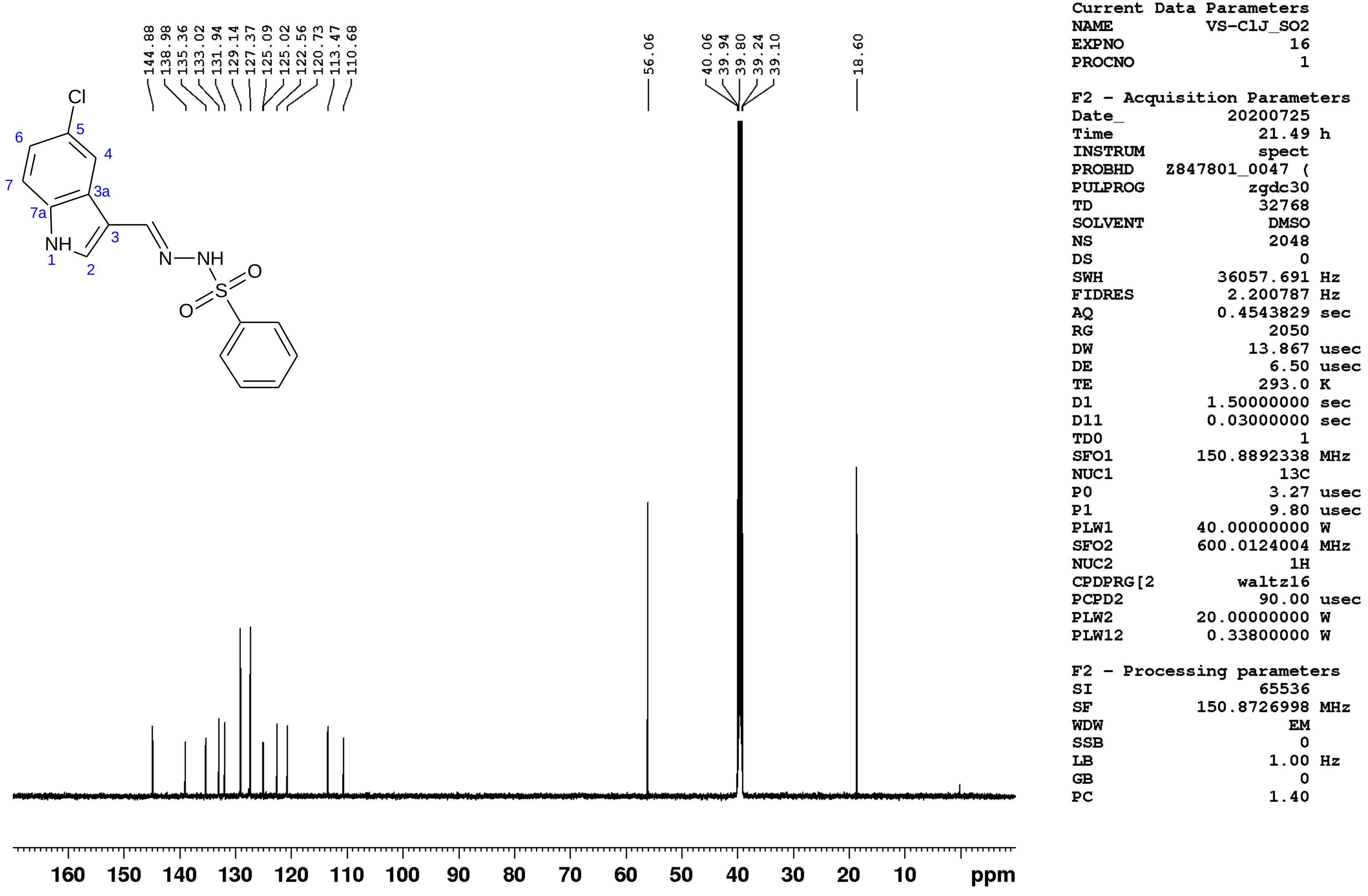

Figure S15. ^{13}C NMR spectrum of *N*'-[(*E*)-(5-chloro-1*H*-indol-3-yl)methylidene]benzenesulfonohydrazide, **1e** in DMSO-d_6

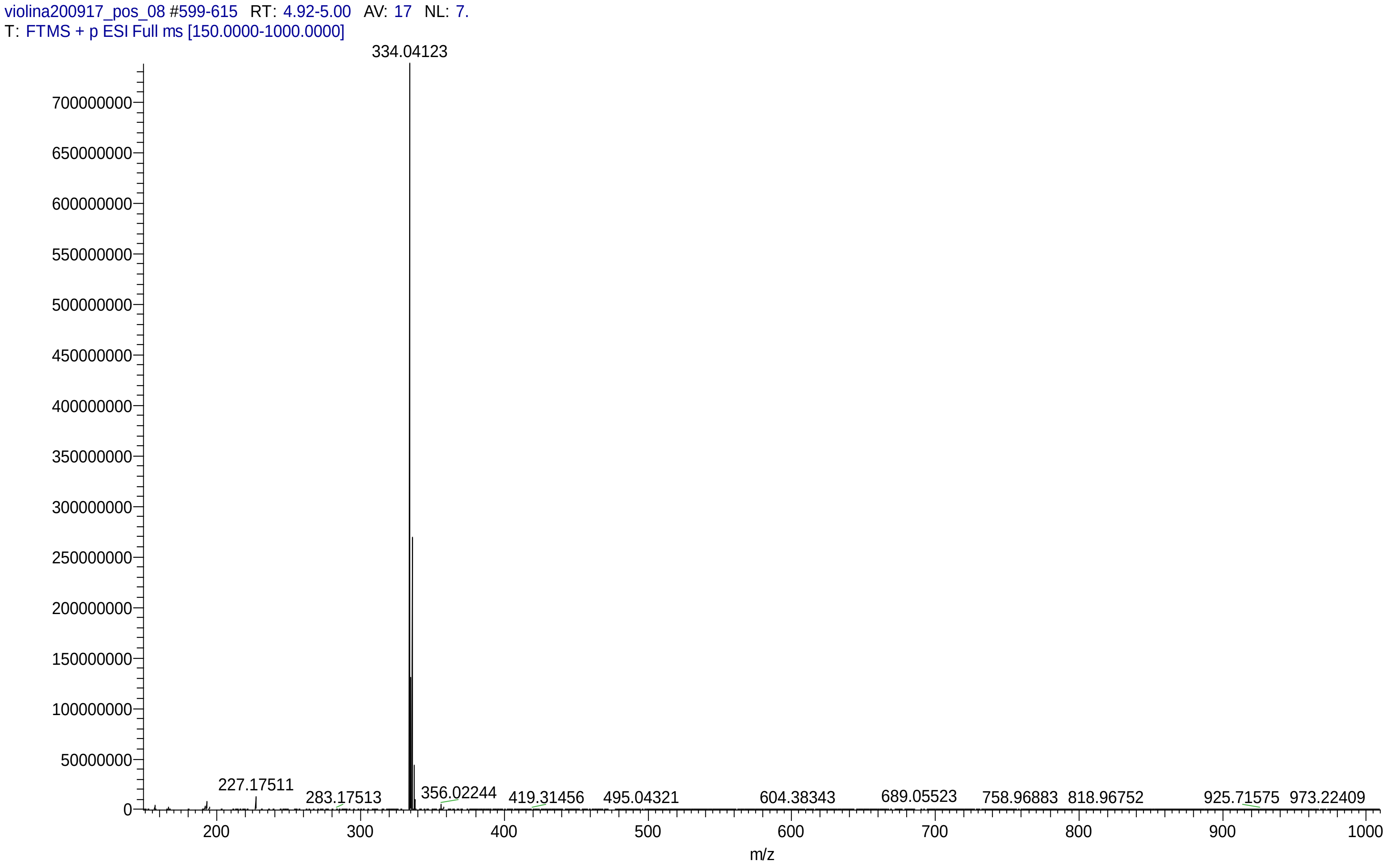

Figure S16. HRMS of *N*-[(*E*)-(5-chloro-1*H*-indol-3-yl)methylidene]benzenesulfonohydrazide, **1e**

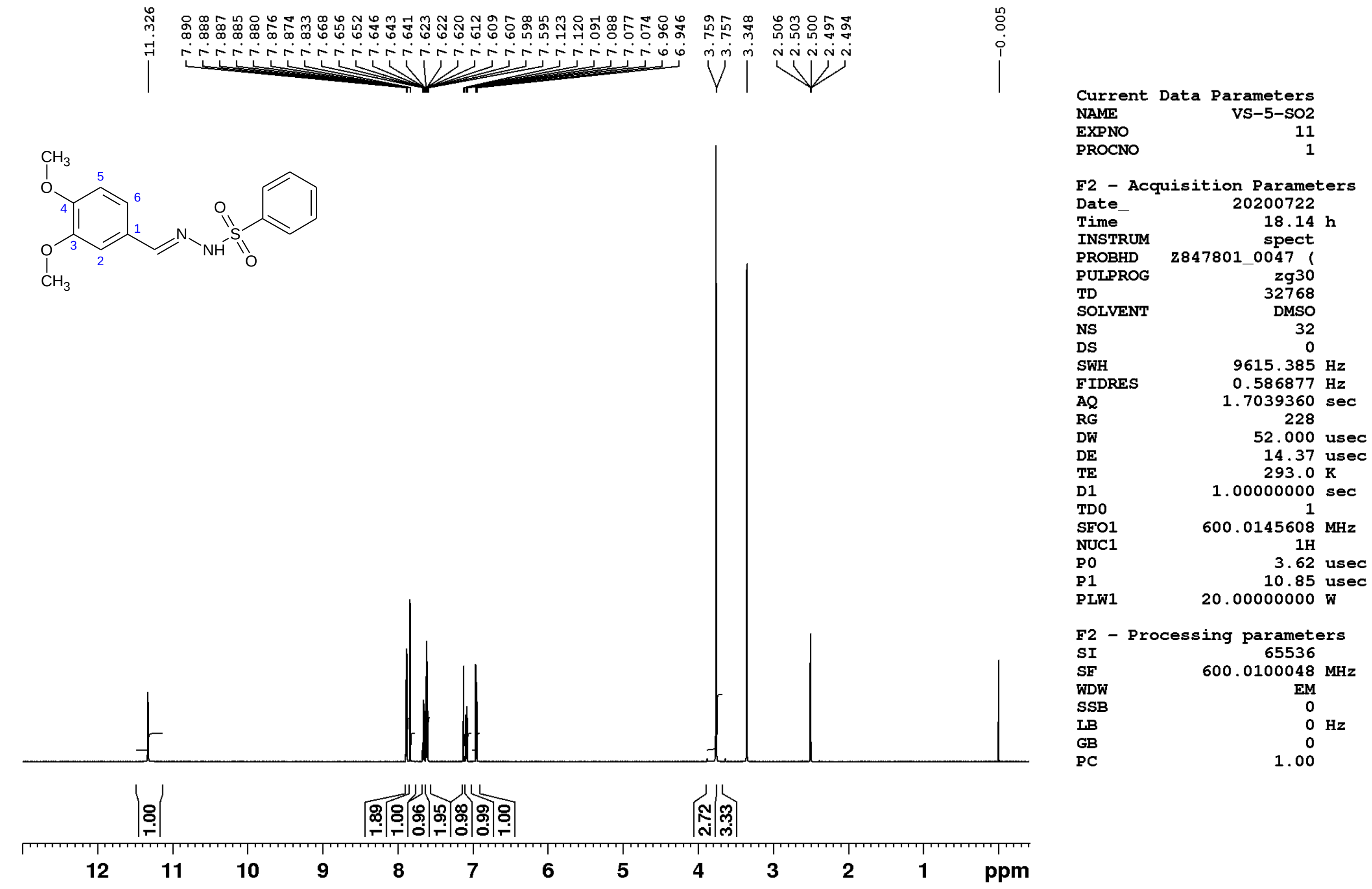

Figure S17. ^{1}H NMR spectrum of *N'*-[(*E*)-(5-chloro-1*H*-indol-3-yl)methylidene]benzenesulfonohydrazide, **1f** in DMSO-d_6

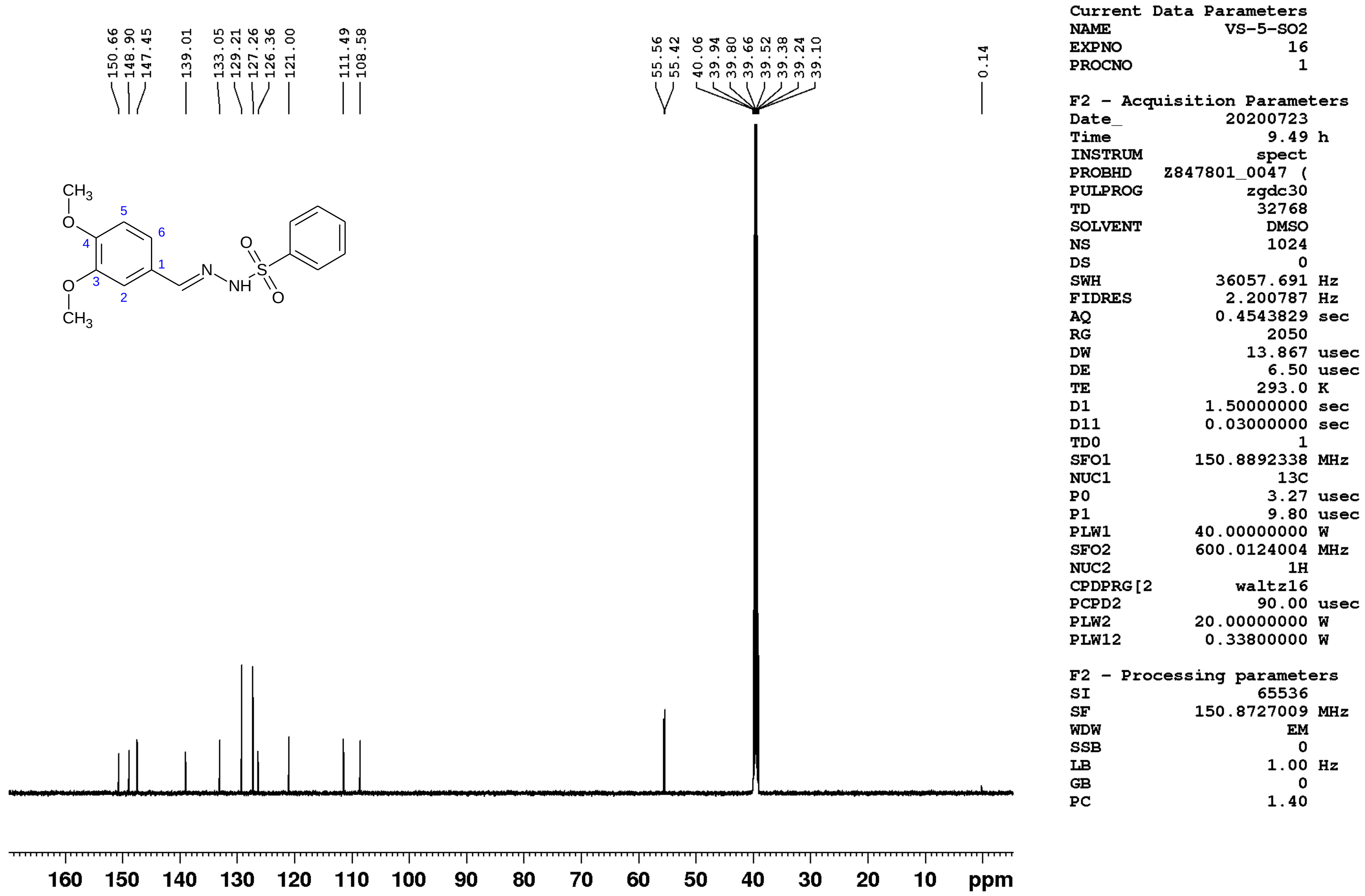

Figure S18. ^{13}C NMR spectrum of *N*'-[(*E*)-(3,4-dimethoxyphenyl)methylidene]benzenesulfonohydrazide, **1f** in DMSO-d_6

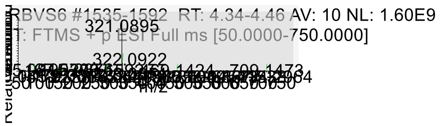

Figure S19. HRMS of *N'*-[(*E*)-(3,4-dimethoxyphenyl)methylidene]benzenesulfonohydrazide, **1f**

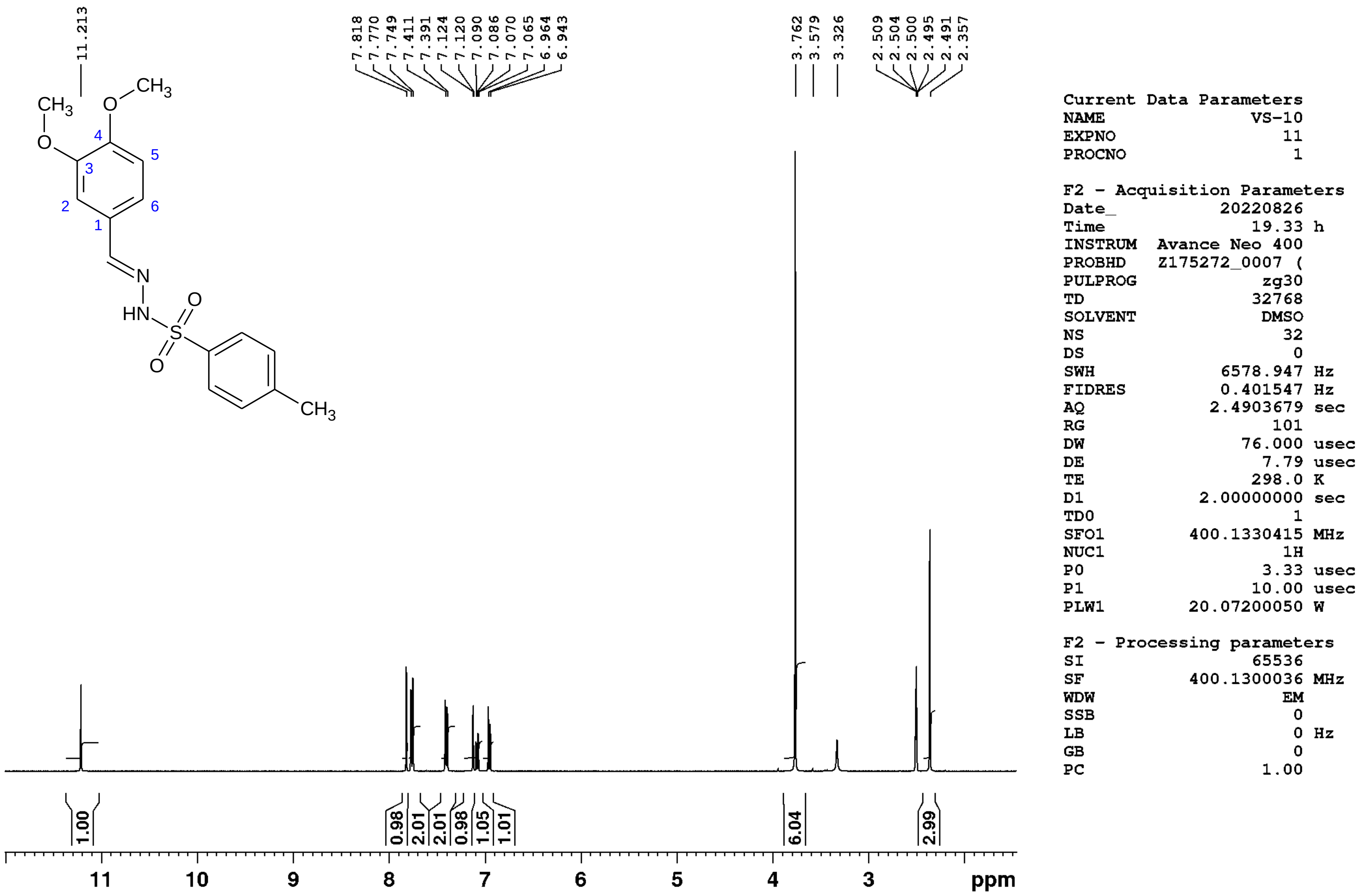

Figure S20. ^{1}H NMR spectrum of *N'*-[(*E*)-(3,4-dimethoxyphenyl)methylidene]-4-methylbenzenesulfonohydrazide, **1g** in DMSO-d_6

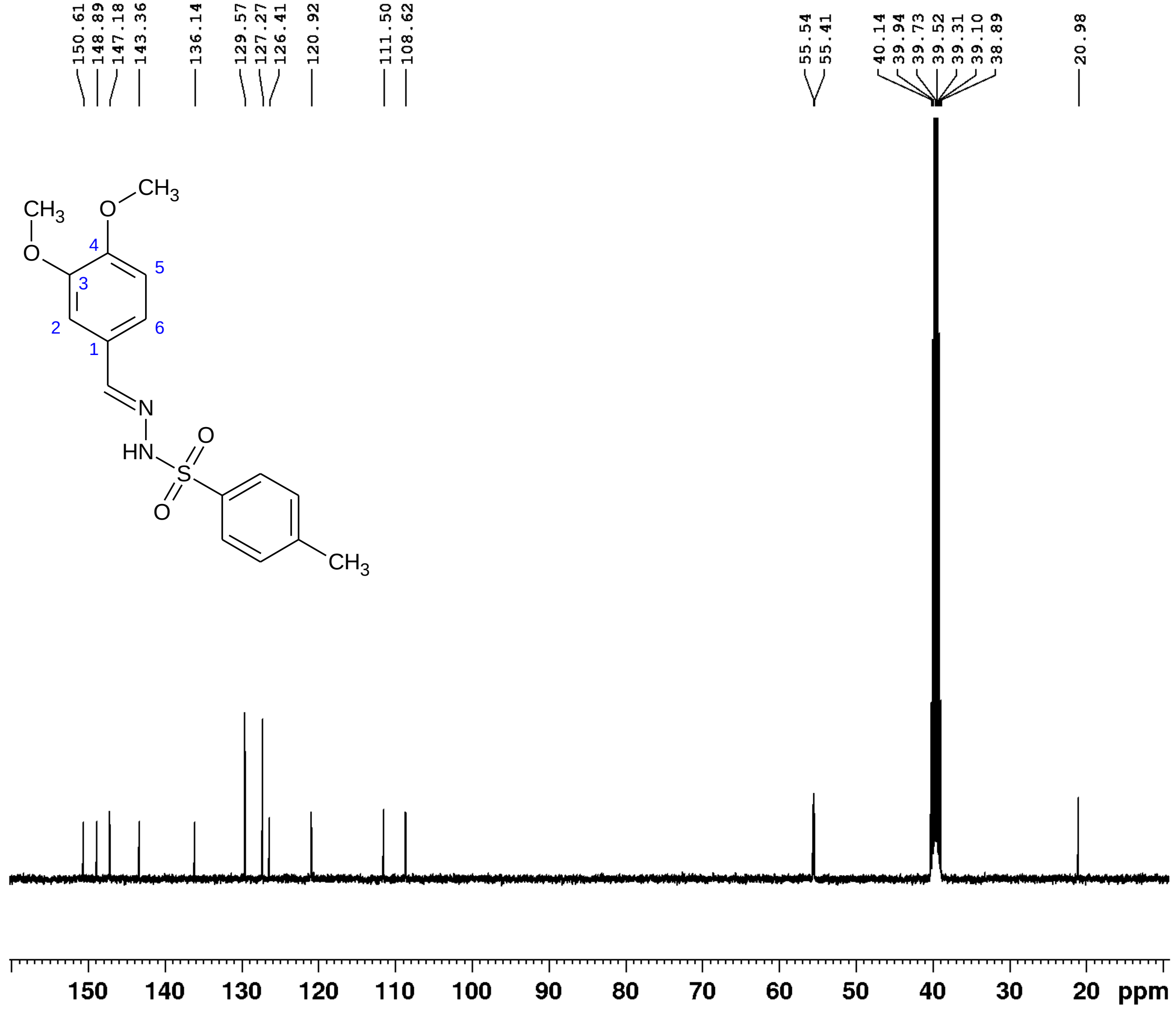


```
Current Data Parameters
NAME                VS-10
EXPNO                  17
PROCNO                  1

F2 - Acquisition Parameters
Date_            20220827
Time                15.13 h
INSTRUM   Avance Neo 400
PROBHD    Z175272_0007 (
PULPROG            zgdc30
TD                  32768
SOLVENT              DMSO
NS                   1024
DS                      0
SWH             23809.523 Hz
FIDRES           1.453218 Hz
AQ              0.6881280 sec
RG                     32
DW                 21.000 usec
DE                   6.50 usec
TE                  298.0 K
D1             1.50000000 sec
D11            0.03000000 sec
TD0                     1
SFO1          100.6238383 MHz
NUC1                  13C
P0                   3.33 usec
P1                  10.00 usec
PLW1          58.46900177 W
SFO2          400.1316008 MHz
NUC2                   1H
CPDPRG[2          waltz16
PCPD2               90.00 usec
PLW2          20.07200050 W
PLW12          0.24781001 W

F2 - Processing parameters
SI                  65536
SF            100.6128172 MHz
WDW                    EM
SSB                     0
LB                   1.00 Hz
GB                      0
PC                   1.40
```

Figure S21. ^{13}C NMR spectrum of *N'*-[(*E*)-(3,4-dimethoxyphenyl)methylidene]-4-methylbenzenesulfonohydrazide, **1g** in DMSO-d_6

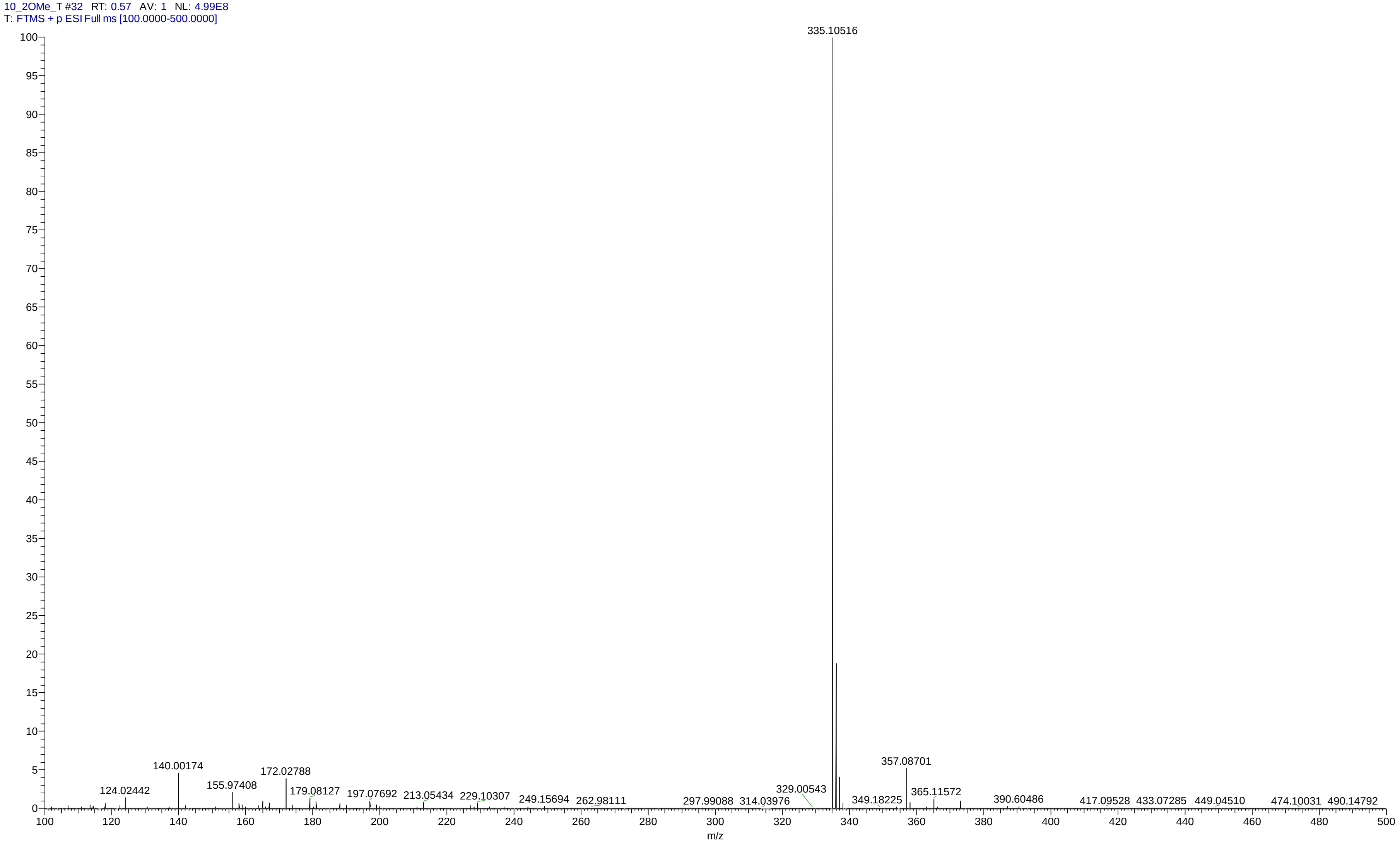

Figure S22. HRMS of *N'*-[(*E*)-(3,4-dimethoxyphenyl)methylidene]-4-methylbenzenesulfonohydrazide, **1g**

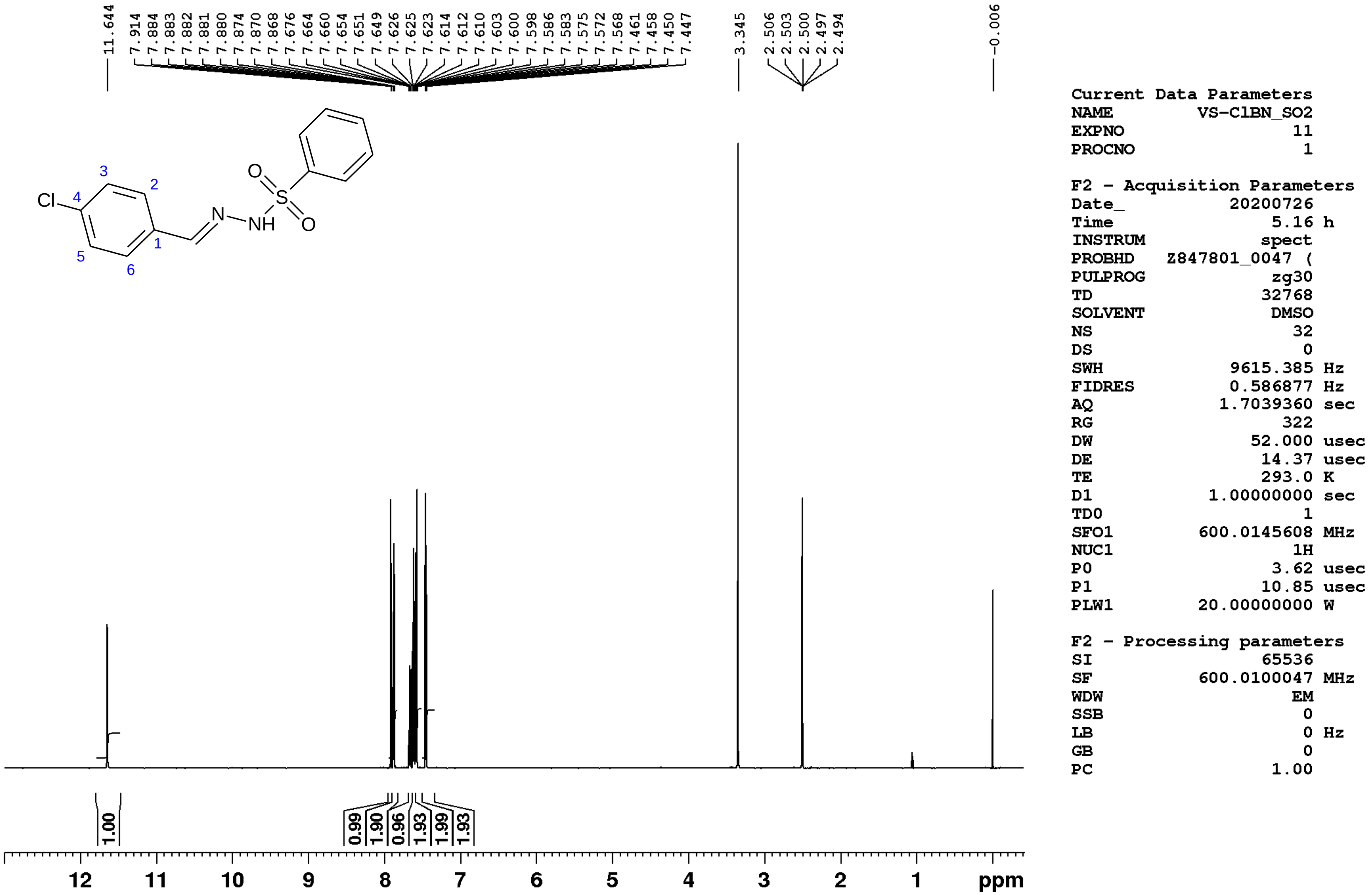

Figure S23. ^{1}H NMR spectrum of *N*'-[(*E*)-(4-chlorophenyl)methylidene]benzenesulfonohydrazide, **1h** in DMSO-d_6

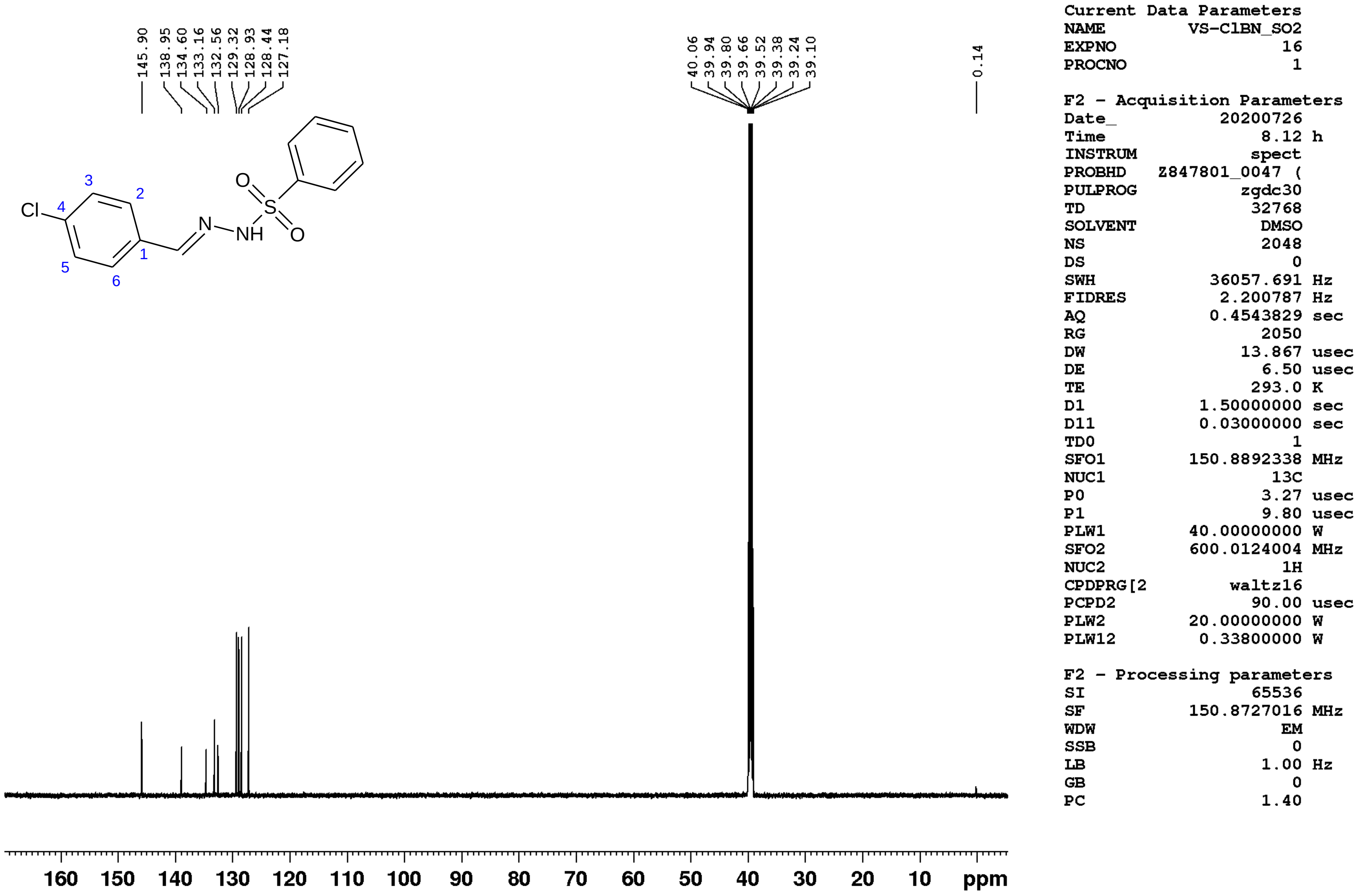

Figure S24. ^{13}C NMR spectrum of *N*'-[(*E*)-(4-chlorophenyl)methylidene]benzenesulfonohydrazide, **1h** in DMSO-d_6

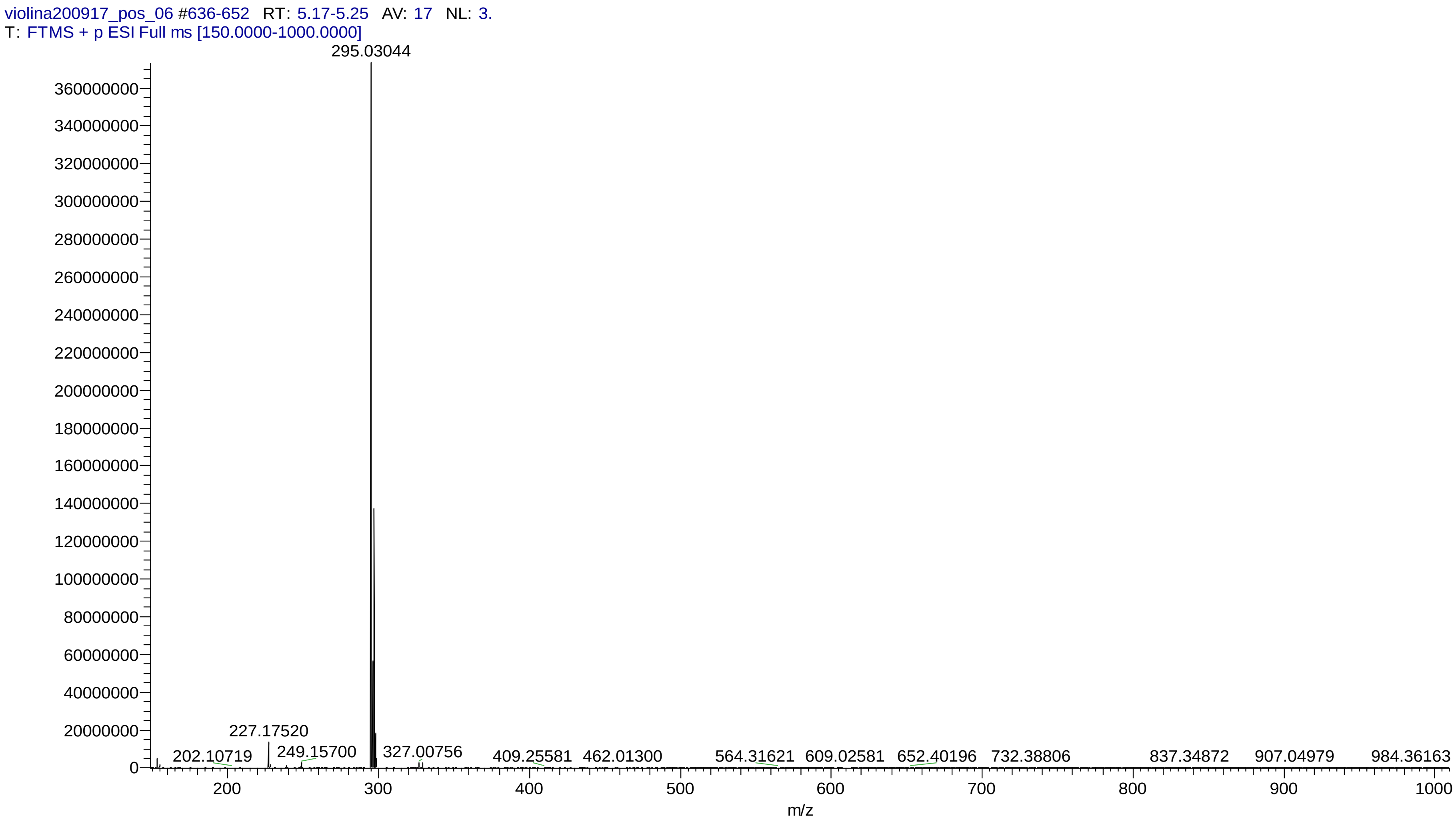

Figure S25. HRMS of *N*'-[(*E*)-(4-chlorophenyl)methylidene]benzenesulfonohydrazide, **1h**

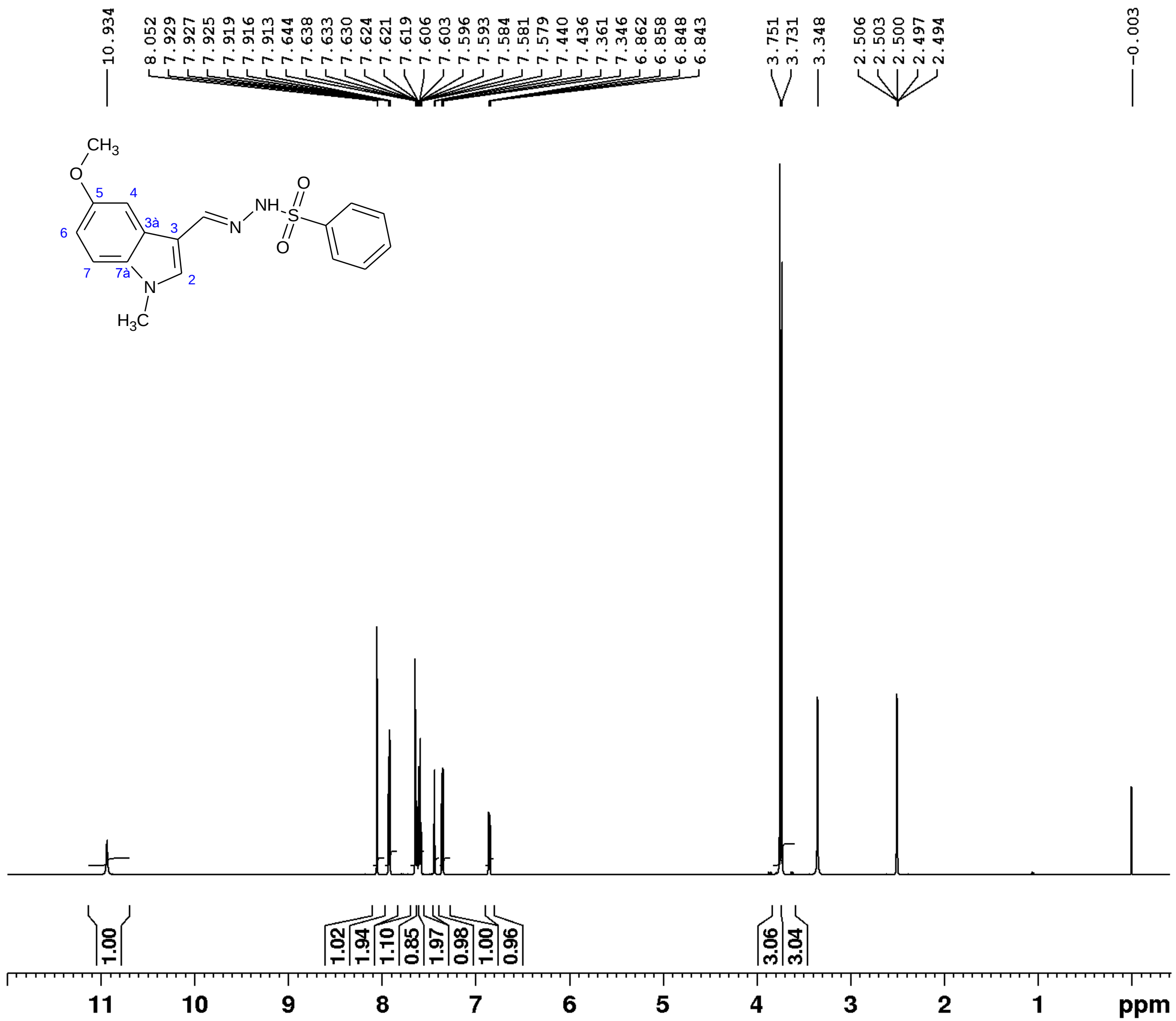

Current Data Parameters
NAME VS-NJ_SO2
EXPNO 11
PROCNO 1

F2 - Acquisition Parameters
Date_ 20200726
Time 1.47 h
INSTRUM spect
PROBHD Z847801_0047 (
PULPROG zg30
TD 32768
SOLVENT DMSO
NS 32
DS 0
SWH 9615.385 Hz
FIDRES 0.586877 Hz
AQ 1.7039360 sec
RG 256
DW 52.000 usec
DE 14.37 usec
TE 293.0 K
D1 1.00000000 sec
TD0 1
SFO1 600.0145608 MHz
NUC1 1H
P0 3.62 usec
P1 10.85 usec
PLW1 20.00000000 W

F2 - Processing parameters
SI 65536
SF 600.0100048 MHz
WDW EM
SSB 0
LB 0 Hz
GB 0
PC 1.00

Figure S26. ^{1}H NMR spectrum of *N'*-[(*E*)-(5-methoxy-1-methyl-1*H*-indol-3-yl)methylidene]benzenesulfonohydrazide, **1i** in DMSO-d_6

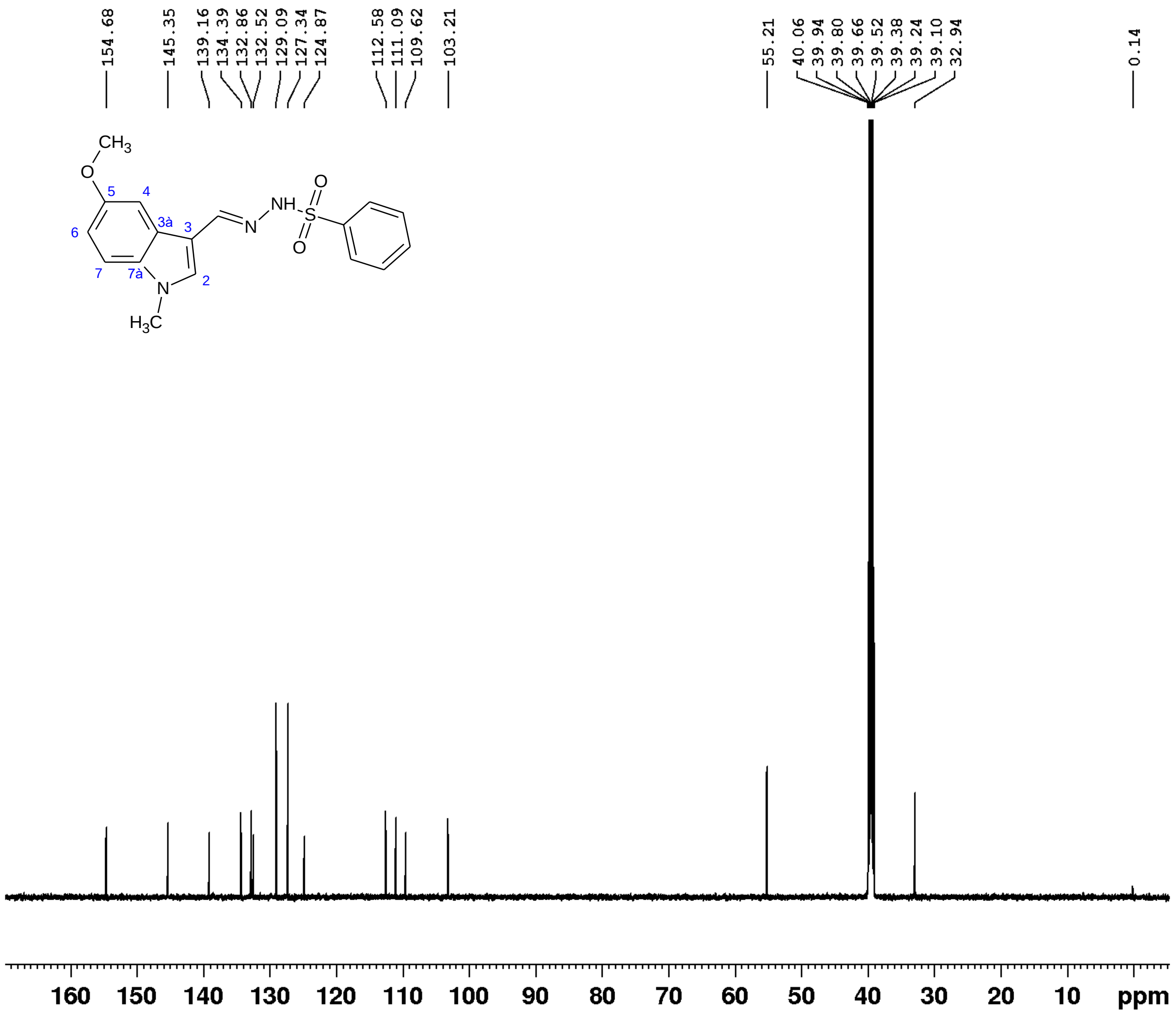


```
Current Data Parameters
NAME            VS-NJ_SO2
EXPNO                  16
PROCNO                  1

F2 - Acquisition Parameters
Date_            20200726
Time                 4.44 h
INSTRUM             spect
PROBHD    Z847801_0047 (
PULPROG            zgdc30
TD                  32768
SOLVENT              DMSO
NS                   2048
DS                      0
SWH             36057.691 Hz
FIDRES           2.200787 Hz
AQ              0.4543829 sec
RG                   2050
DW                 13.867 usec
DE                   6.50 usec
TE                  293.0 K
D1             1.50000000 sec
D11            0.03000000 sec
TD0                     1
SFO1          150.8892338 MHz
NUC1                  13C
P0                   3.27 usec
P1                   9.80 usec
PLW1          40.00000000 W
SFO2          600.0124004 MHz
NUC2                   1H
CPDPRG[2          waltz16
PCPD2               90.00 usec
PLW2          20.00000000 W
PLW12          0.33800000 W

F2 - Processing parameters
SI                  65536
SF            150.8727015 MHz
WDW                    EM
SSB                     0
LB                   1.00 Hz
GB                      0
PC                   1.40
```

Figure S27. ^{13}C NMR spectrum of *N'*-[(*E*)-(5-methoxy-1-methyl-1*H*-indol-3-yl)methylidene]benzenesulfonohydrazide, **1i** in DMSO-d_6

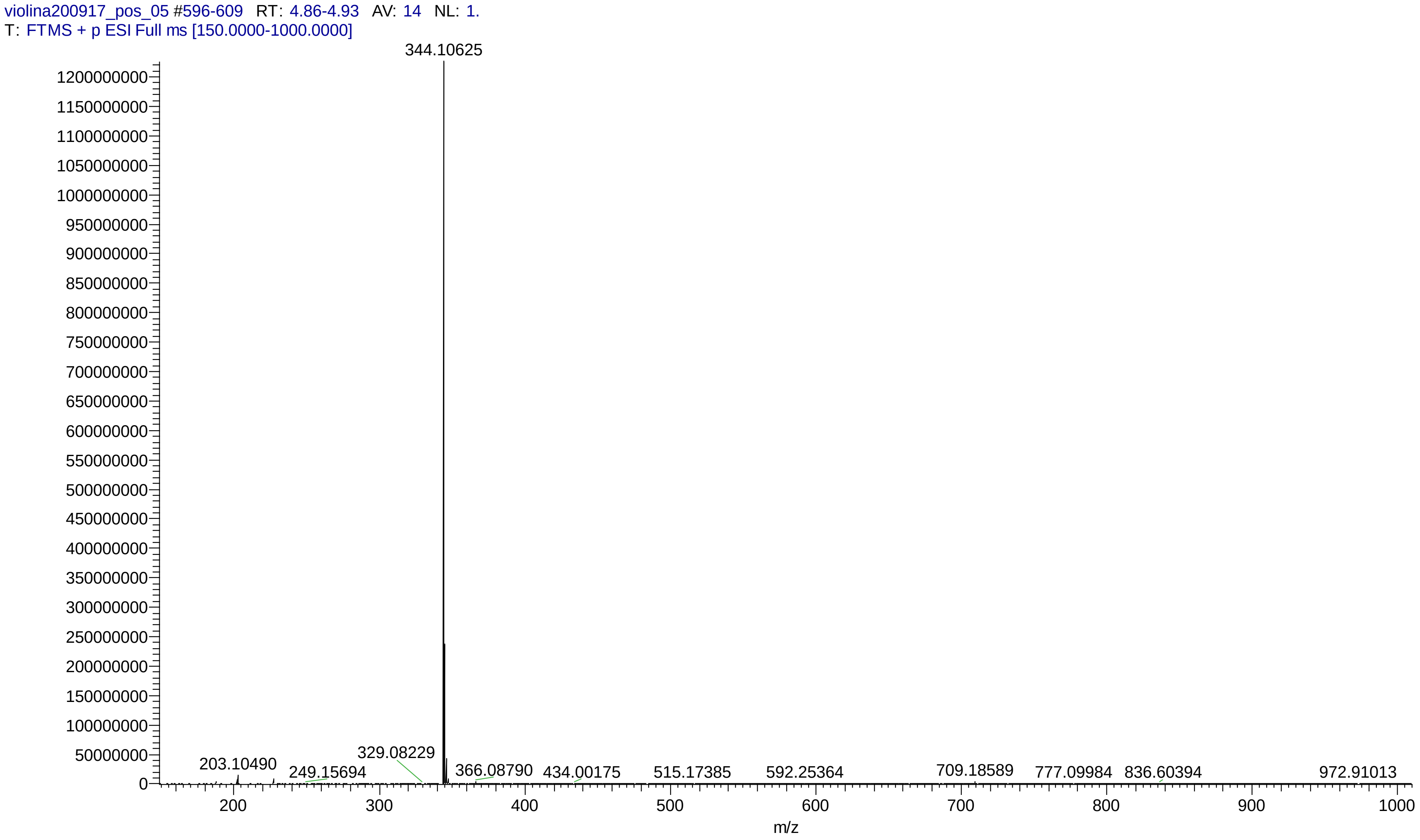

Figure S28. HRMS of *N'*-[(*E*)-(5-methoxy-1-methyl-1*H*-indol-3-yl)methylidene]benzenesulfonohydrazide, **1i**

Table S1. Values of the descriptors relevant for the cytotoxic activity on MCF-7 and MDA-MB-468 of aryl-sulfonyl hydrazones from the training set and the newly designed compounds.Compounds **3a–o** are collected from Senkardes et al. [12], compounds **5a–k** – from Gaur et al. [13], compounds **1a–i** are newly designed, synthesized and tested in the present study. *LE* stands for Ligand Efficiency. The anticancer activities of the compounds are measured on human breast adenocarcinoma cell line MCF-7 (n = 26) and on the TNBC cell line MDA-MB-468 (n = 11). *morph* is a user-defined indicator differentiating the two subsets in the training set; *SaaaC_acnt* accounts for the number of aromatic *aaaC*-atoms in the molecule; *SaaN_acnt* corresponds to the number of aromatic *aaN*-atoms; *ka1* is first order kappa alpha shape index; *nelem* is the number of chemical elements in the molecule and *nvx* accounts for the number of graph vertices. .

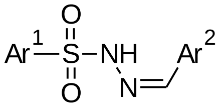

Original ID	Ar1	Ar2	*LE* MCF-7	*LE* MDA-MB-468	*morph*	*SaaaC_acnt*	*SaaN_acnt*	*ka1*	*nelem*	*nvx*
**3a**	4-methylphenyl	2,2-difluoro-1,3-bezodioxol-5-yl	0.174		0	0	0	16.6506	6	24
**3b**	“	4-bromothiophen-2-yl	0.203		0	0	0	14.5177	6	19
**3c**	“	4-phenylthiophen-2-yl	0.189		0	0	0	16.6989	5	24
**3d**	“	4-fluoro-3-phenoxyphenyl	0.121		0	0	0	18.9073	6	27
**3e**	“	2-chloro-3-(trifluoromethyl)phenyl	0.148		0	0	0	18.4388	7	24
**3f**	“	4-fluoro-3-methoxyphenyl	0.207		0	0	0	16.2834	6	22
**3g**	“	4-methoxy-3-nitrophenyl	0.143		0	0	0	18.0447	5	24
**3h**	“	3-phenyl-1*H*-pyrazol-4-yl	0.141		0	0	1	16.2549	5	24
**3i**	“	5-bromo-2-methoxyphenyl	0.170		0	0	0	16.8241	6	22
**3j**	“	4-fluoro-2-(trifluoromethyl)phenyl	0.148		0	0	0	18.0841	6	24
**3k**	“	2-chloro-3-methoxyphenyl	0.197		0	0	0	16.6373	6	22
**3l**	“	2-chloro-6-methylphenyl	0.180		0	0	0	15.6942	6	21
**3m**	“	6-bromopyridin-2-yl	0.176		0	0	0	15.6942	6	21
**3n**	“	1-methyl-1*H*-pyrrol-2-yl	0.207		0	0	0	13.6666	5	19
**3o**	“	2-(trifluoromethoxy)phenyl	0.166		0	0	0	18.1136	6	24
**5a**	4-methoxyphenyl	1-(4-morpholinylethyl)-1*H*-indol-3-yl	0.136	0.141	1	2	0	21.8178	5	31
**5b**	4-methylphenyl	“	0.140	0.146	1	2	0	20.8944	5	30
**5c**	phenyl	“	0.143	0.149	1	2	0	19.9349	5	29
**5d**	4-fluorophenyl	“	0.150	0.161	1	2	0	20.8271	6	30
**5e**	4-nitrophenyl	“	0.128	0.138	1	2	0	22.5503	5	32
**5f**	4-chlorophenyl	“	0.163	0.170	1	2	0	21.1731	6	30
**5g**	4-trimethylphenyl	“	0.126	0.137	1	2	0	23.7866	5	33
**5h**	2-naphthyl	“	0.116	0.110	1	4	0	25.6092	5	39
**5i**	5-quinolyl	“	0.105	0.115	1	4	1	25.5429	5	39

**5j**	methylphenyl	“	0.140	0.143	1	2	0	20.8944	5	30
**5k**	diphenyl	“	0.136	0.128	1	2	0	23.5948	5	35
Cisplatin[1]			0.931							
Doxorubicin[2]			0.185	0.182						
**1a**	phenyl	5-methoxy-1H-indole-3-yl	0.271	0.240	0	2	0	15.5709	5	23
**1b**	4-methylphenyl	5-methoxy-1H-indole-3-yl	0.254	0.211	0	2	0	16.5347	5	24
**1c**	phenyl	1-acetyl-1H-indole-3-yl	0.252	0.197	0	2	0	16.2549	5	24
**1d**	4-methylphenyl	1-acetyl-1H-indole-3-yl	0.230	0.167	0	2	0	17.2207	5	25
**1e**	phenyl	5-chloro-1H-indole-3-yl	0.286	0.275	0	2	0	14.927	6	22
**1f**	phenyl	3,4-dimethoxyphenyl	0.237	0.162	0	0	0	16.3129	5	22
**1g**	4-methylphenyl	3,4-dimethoxyphenyl	0.185	0.143	0	0	0	17.2963	5	23
**1h**	phenyl	4-chlorophenyl	0.200	0.221	0	0	0	13.735	6	19
**1i**	phenyl	1-methyl-1H-indole-3-yl	0.158	0.178	0	2	0	16.5347	5	24

[1] [12]; [2] [13].
